# *Mus musculus* papillomavirus 1 is a key driver of skin cancer development upon immunosuppression

**DOI:** 10.1111/ajt.16358

**Published:** 2020-11-03

**Authors:** Sonja Dorfer, Katharina Strasser, Georg Schröckenfuchs, Michael Bonelli, Wolfgang Bauer, Harald Kittler, Christophe Cataisson, Michael B. Fischer, Beate M. Lichtenberger, Alessandra Handisurya

**Affiliations:** ^1^ Department of Dermatology Medical University of Vienna Vienna Austria; ^2^ Department of Internal Medicine III Medical University of Vienna Vienna Austria; ^3^ Laboratory of Cancer Biology and Genetics National Institutes of Health National Cancer Institute Bethesda MD USA; ^4^ Department of Transfusion Medicine Medical University of Vienna Vienna Austria

**Keywords:** basic (laboratory) research / science, dermatology, infectious disease, immunosuppression / immune modulation, animal models: murine, infection and infectious agents ‐ viral, cancer / malignancy / neoplasia: skin ‐ nonmelanoma, immunosuppressant ‐ calcineurin inhibitor: cyclosporine A (CsA)

## Abstract

Epidemiological and experimental data implicate cutaneous human papillomavirus infection as co‐factor in the development of cutaneous squamous cell carcinomas (cSCCs), particularly in immunocompromised organ transplant recipients (OTRs). Herein, we established and characterized a skin cancer model, in which *Mus musculus* papillomavirus 1 (MmuPV1) infection caused cSCCs in cyclosporine A (CsA)‐treated mice, even in the absence of UV light. Development of cSCCs and their precursors were observed in 70% of MmuPV1‐infected, CsA‐treated mice on back as well as on tail skin. Immunosuppression by systemic CsA, but not UV‐B irradiation, was a prerequisite, as immunocompetent or UV‐B–irradiated mice did not develop skin malignancies after infection. In the virus‐driven cSCCs the MmuPV1‐E6/E7 oncogenes were abundantly expressed, and transcriptional activity and productive infection demonstrated. MmuPV1 infection induced the expression of phosphorylated H2AX, but not degradation of proapoptotic BAK in the cSCCs. Transfer of primary cells, established from a MmuPV1‐induced cSCC from back skin, into athymic nude mice gave rise to secondary cSCCs, which lacked viral DNA, demonstrating that maintenance of the malignant phenotype was virus independent. This papillomavirus‐induced skin cancer model opens future investigations into viral involvement, pathogenesis, and cancer surveillance, aiming at understanding and controlling the high incidence of skin cancer in OTRs.

## Abstract 2

This murine model demonstrates the synergy of *Mus musculus* papillomavirus 1 skin infection and systemic immunosuppression in the development of cutaneous squamous cell carcinomas, even in the absence of UV‐B light.

**Abbreviations**
CPD: cyclobutane pyrimidine dimers
CsA: cyclosporine A
cSCC: cutaneous squamous cell carcinoma
EV: Epidermodysplasia verruciformis
FCS: fetal calf serum
HE: hematoxylin/eosin
HPV: human papillomavirus
IHC: immunohistochemistry
MmuPV1: *Mus musculus* papillomavirus 1
OTR: organ transplant recipient
pan‐CK: pan‐cytokeratin
PsV: pseudovirion
PsV‐NA: PsV‐neutralization assay
SD: standard deviation
TCR: T cell receptor
tg: transgenic
UV: ultraviolet
γH2AX: gamma histone 2AX

## 1 INTRODUCTION

Cutaneous squamous cell carcinoma (cSCC) represents the second most common type of skin cancer worldwide. A meta‐analysis estimated the numbers of new cases in the United States White population in 2012 to be between 186 000 and 419 000, with increasing incidence.^1^ As cSCCs are not specifically disclosed in national cancer registries, the exact numbers are unknown.^1^, ^2^

Organ transplant recipients (OTRs) are particularly vulnerable with up to 250‐fold higher cSCC rates compared to the general population, and 40% of the afflicted develop skin malignancies within 15 years after transplantation.^3^ Recently, an 812 per 100 000 person‐years incidence was estimated in this population in the United States.^4^ Aside from common risk factors, such as high cumulative ultraviolet (UV) exposure, genetic predisposition, and chronic inflammation, the cSCC rates in OTRs are influenced by intensity, duration, and type of immunosuppression.^5^ Commonly used immunosuppressants include calcineurin inhibitors (cyclosporine A [CsA], tacrolimus), mammalian target of rapamycin inhibitors (rapamycin/sirolimus, everolimus), and antimetabolites (azathioprine, mycophenolate mofetil).

Oncogenic mucosal alpha‐HPVs can persistently infect mucosa of the anogenital and upper aerodigestive tract to induce cancer at these sites. For keratinocyte cancers, the role of cutaneous HPVs, in particular of genus beta (beta‐HPVs), has been a matter of debate for decades.^6^, ^7^, ^8^, ^9^ In the rare genodermatosis *Epidermodysplasia verruciformis* (EV), certain genus beta‐HPVs are responsible for the development of cSCCs on sun‐exposed areas, which contain high viral loads and actively transcribed E6/E7 oncogenes.^10^ OTRs have a higher risk for human papillomavirus (HPV) infections due to suppressed cell‐mediated immunity. Numerous epidemiologic studies have implicated beta‐HPV skin infection as risk factor for cSCCs in this population, based on viral positivity and on seropositivity at the time of transplantation,^11^, ^12^, ^13^ although others argue against a causal role, given that viral DNA is not present in all cancer cells and can also be found on skin surfaces in the general population.^8^, ^9^ The carcinogenic potential of beta‐HPVs was further demonstrated in several animal models. For instance, transgenic (tg) beta‐HPV8 mice, which expressed the viral genome in the skin, developed cSCCs without exposure to UV light or chemical carcinogens.^14^ The carcinogenic synergism between the viral oncogenes and the major causative factor for skin cancer development, UV light, was shown in tg beta‐HPV mice and in *Mastomys natalensis* or *Mus musculus* papillomavirus (MmuPV1)‐infected rodents, which developed cSCCs after (long‐term) UV irradiation.^15^, ^16^, ^17^, ^18^, ^19^, ^20^ In contrast to alpha‐HPVs, which can immortalize cells and induce genomic instability after integration, beta‐HPV oncogenes were shown to compromise DNA damage repair and inhibit apoptosis,^3^ allowing accumulation of mutations in keratinocytes. Hence, beta‐HPVs are thought to contribute as initial tumor promoter and progression factors to carcinogenesis rather than to maintenance of malignancy (“hit‐and‐run‐mechanism”).^6^, ^10^

Herein, we addressed the questions, whether MmuPV1 infection per se can induce cSCC development in the absence of UV light and which role an intact immune system plays in preventing tumor development. Hence, we aimed at establishing and characterizing a laboratory mouse model, in which experimental MmuPV1 infection caused cSCCs in CsA‐immunocompromised mice.

## 2 MATERIALS AND METHODS

### 2.1 Ethics statement

Animal studies were approved by the ethics committee of the Medical University of Vienna, Austria, and the Austrian Federal Ministry of Science and Research (BMWFW‐66.009/0304‐V/3b/2019, BMWFW‐66.009/0228‐WF/V/3b/2016) and performed in full compliance with the institutional guidelines.

### 2.2 MmuPV1‐induced skin cancer model

Immunocompetent, female FVB/NCrl mice aged 4‐5 weeks were obtained from Charles River Laboratories (Sulzfeld, Germany), immunodeficient NMRI‐Foxn1^nu/nu^ mice bred in‐house. A schematic representation of the experimental setup is shown in Figure S1.

FVB/NCrl mice (n = 40) were infected with 1 × 10^10^ MmuPV1 virions per site on the skin of back and tail.^21^ The same number of littermates served as uninfected controls. CsA was administered subcutaneously to 30 MmuPV1‐infected and 30 uninfected mice at a dose of 75 mg/kg body weight five times per week, starting 1 week prior to MmuPV1 inoculation, for a total of 11 weeks. The immunosuppressive state was maintained by CsA administration three times per week until week 30 postinfection. A subgroup (n = 25 MmuPV1‐infected, n* =* 25 uninfected) was UV‐B irradiated at a dose of 120 mJ/cm^2^ three times per week employing a Waldmann TP‐4 UV lamp (Herbert Waldmann GmbH & Co. KG, Villingen‐Schwenningen, Germany), starting 1 week prior to inoculation. The UV‐B dose was increased weekly by 16.5 mJ/cm^2^ until the final dose of 450 mJ/cm^2^ was reached at week 20 postinfection.^17^ Irradiation was continued until week 30 postinfection to a cumulative dose of 30 J/cm^2^.

Skin tissues were procured from the inoculation sites at week 30 postinfection, regardless whether visible lesions had evolved. Some tumors were collected prior to the end of the experiment for ethical reasons, when tumors reached impairing sizes or at signs of sickness. The back tumor area in mm^2^ and the tail tumor length in mm were determined employing ImageJ software.

### 2.3 Hematoxylin/eosin (HE) staining and immunohistochemistry (IHC)

Skin sections taken from the inoculation sites and the draining inguinal lymph nodes were stained with HE for subsequent evaluation by a pathologist blinded to the experimental conditions or used for IHC (Table S1). The MmuPV1‐L1/L2‐specific polyclonal immune serum was generated by immunization of a New Zealand White rabbit with MmuPV1‐L1/L2 pseudovirions (PsVs) in a four‐dose regimen at week 0‐2‐4‐8 (Eurogentec, Seraing, Belgium). Images were digitalized using an Aperio slide scanner (Leica Biosystems, Nussloch, Germany). The numbers of immunopositive cells (per mm^2^) and the immunopositive area (in %) present in the entire skin or lymph node tissue specimens were scored independently by three authors (S.D., K.S., and G.S.) employing ImageJ.

### 2.4 RNA in situ hybridization

Single molecule RNA in situ hybridization for the MmuPV1‐E6/E7 mRNA was performed employing the RNAscope *MusPV‐E6‐E7* probe (Advanced Cell Diagnostics, Newark, CA). RNA integrity was verified with the endogenous control probe *Mm‐PPIB*, background staining evaluated using the negative control probe specific for the bacterial *DapB* gene. Brightfield images were acquired using the Aperio slide scanner (Leica Biosystems).

### 2.5 Quantification of MmuPV1 genomic DNA and E1^E4 splice transcripts

Total RNA and genomic DNA were purified from the same tissue specimens using TRI reagent (Sigma‐Aldrich). MmuPV1 DNA copy numbers were normalized to the housekeeping gene gamma‐actin and quantified according to standard curves with known amounts of the religated viral genome.^21^, ^22^ MmuPV1‐E1^E4 spliced transcripts, a marker for infectivity and viral transcription, were determined as described previously^21^ and the results were normalized to the endogenous control beta‐actin (Thermo Fisher Scientific). The *n*‐fold changes were calculated using the comparative CT (ΔΔCT) method.^23^ MmuPV1‐E1‐specific primers (forward: 5′‐AATCGGCAAAGGCTACAC‐3‘, reverse: 5'‐AACGAGTCTGGTGACAAC‐3‘) were employed for conventional PCR.

### 2.6 In vivo passaging of cSCC‐derived primary cells

A primary cell line was established from a cSCC of a MmuPV1‐infected, CsA‐/UV‐B–treated mouse. Briefly, a 8 mm^3^ piece of the tumor was minced with a scalpel under sterile conditions and transferred to a 9.2 cm^2^ tissue culture dish containing RPMI 1640, 10% fetal calf serum (FCS) and 2% penicillin‐streptomycin. Outgrowing cells were serially passaged in RPMI 1640, 5% FCS, 1% penicillin‐streptomycin.

250,000‐500,000 cSCC cells were administered intradermally into the back and both flanks of two NMRI‐Foxn1^nu/nu^ mice. Animals were monitored for lesion development until 30 days postinoculation.

### 2.7 Particle‐ELISA and PsV‐neutralization assay (NA)

MmuPV1‐specific antibodies in mouse sera (diluted 1:100) were assessed by particle‐ELISA employing native virions as the antigen.^24^ Neutralizing antibodies were determined by PsV‐NA employing MmuPV1‐SEAP PsVs composed of the MmuPV1 capsid proteins L1/L2 and the reporter pYSEAP, encoding for secreted alkaline phosphatase.^25^ The IC_50_ titer is defined as the highest serum dilution showing at least 50% reduction in SEAP signal compared to the preimmune sera or in the absence of immune sera.

### 2.8 Statistical analyses

Analyses were performed using GraphPad Prism 8. Data represent mean±standard deviation (SD). Differences between groups were analyzed using the nonparametric Kruskal–Wallis test with the Dunn's posttest. A *P*‐value of <.05 was considered statistically significant.

## 3 RESULTS

### 3.1 MmuPV1 infection causes cSCC development in CsA‐treated FVB/NCrl mice

**FIGURE 1:**
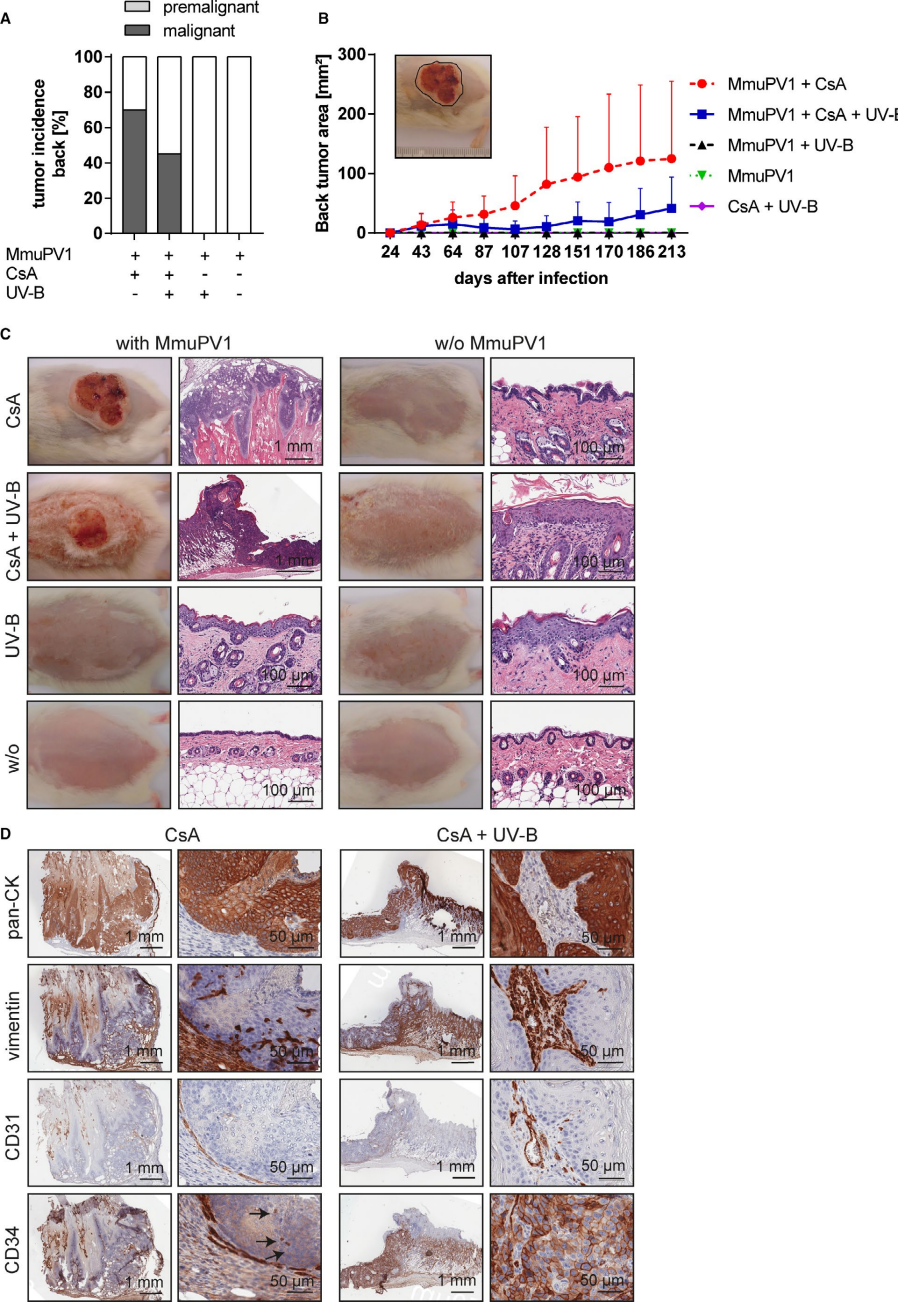
Tumor incidence after experimental MmuPV1 skin infection. (A) Tumor incidence on back skin in MmuPV1‐infected mice at week 30 postinfection. None of the uninfected mice developed skin tumors. (B) Time course of tumor outgrowth on back skin. Tumor area is given in mm^2^. (C) Representative mouse of each experimental group with corresponding HE image. Left panel, MmuPV1‐infected mice; right panel, uninfected mice. (D) Pan‐cytokeratin, vimentin, CD31, and CD34 stainings of cSCCs of back skin. One representative MmuPV1‐infected, CsA‐treated (far‐left and left panels) and MmuPV1‐infected CsA‐/UV‐B–treated (far‐right and right panels) mouse is depicted. Overview and higher magnification of the same tumors are shown

Experimental infection with MmuPV1 of back skin (Figure 1A‐C) resulted in cSCC development in the majority (70%; 7/10) of CsA‐immunosuppressed mice. Tumor outgrowth started around day 30 and continued without signs of regression until week 30 postinfection. The tumors were large, with mean sizes of 124.8 (±130.6) mm^2^ at procurement. Concomitant UV‐B irradiation of MmuPV1‐infected, CsA‐treated animals allowed for cSCC development in 45.0% (9/20) and the tumors had smaller mean sizes of 41.1 (±53.2) mm^2^. In the absence of CsA, tumors were not observed in MmuPV1‐infected mice (0%; 0/10), even though a subset of the animals were concomitantly UV‐B irradiated (0%; 0/5).

On the tail skin (Figure S2A‐C), invasive cSCCs were found in 30.0% (3/10) and preinvasive carcinomas in situ in 40.0% (4/10) of MmuPV1‐infected, CsA‐treated mice with mean tumor lengths of 14.3 (±6.0) mm. MmuPV1‐infected, CsA‐/UV‐B–treated mice had invasive and preinvasive cSCCs in 75.0% (15/20) and 15.0% (3/20), respectively, with comparable mean tumor lengths of 13.5 (±5.2) mm. UV‐B irradiation of immunocompetent, virus‐infected mice resulted in a 5.6 mm long cSCC on the tail skin of one mouse (20%; 1/5) and allowed for the persistence of benign papillomas in 40% (2/5). Small, transient papillomas were observed in 60% (3/5) of MmuPV1‐infected mice, which completely regressed within 2 months postinfection.

The skin cancers were dependent on MmuPV1 infection, as uninfected mice (0%; 0/40) did not develop tumors on back or tail skin, regardless of treatment with CsA (0%; 0/10), UV‐B (0%; 0/5) or both (0%; 0/20). The immunosuppressive effect of CsA was a prerequisite for outgrowth of cSCCs and the differences between MmuPV1‐infected, CsA‐treated and MmuPV1‐infected, non‐CsA–treated groups were statistically significant (*P* = .0193 and *P* = .0043 for tumor sizes on back and tail skin, respectively, at week 30 postinfection).

The cSCC cells stained positive for pan‐cytokeratin, confirming the epithelial nature of the malignancies. Vimentin staining was found in tumor stroma and a small subset of the malignant tumor cells of back and tail skin. Stainings of the pan‐endothelial marker CD31 in peri‐ and intratumoral vessels showed vascular invasion into the tumors. Endothelial cells, fibrocytes, myofibroblasts and peritumoral stroma were CD34 immunopositive. The vast majority of the tumors were CD34‐negative; however, in a subset of cSCCs of the back, some CD34‐positive tumor cells were observed (Figure 1D).

### 3.2 MmuPV1 is present and transcriptionally active in the cSCCs

**FIGURE 2:**
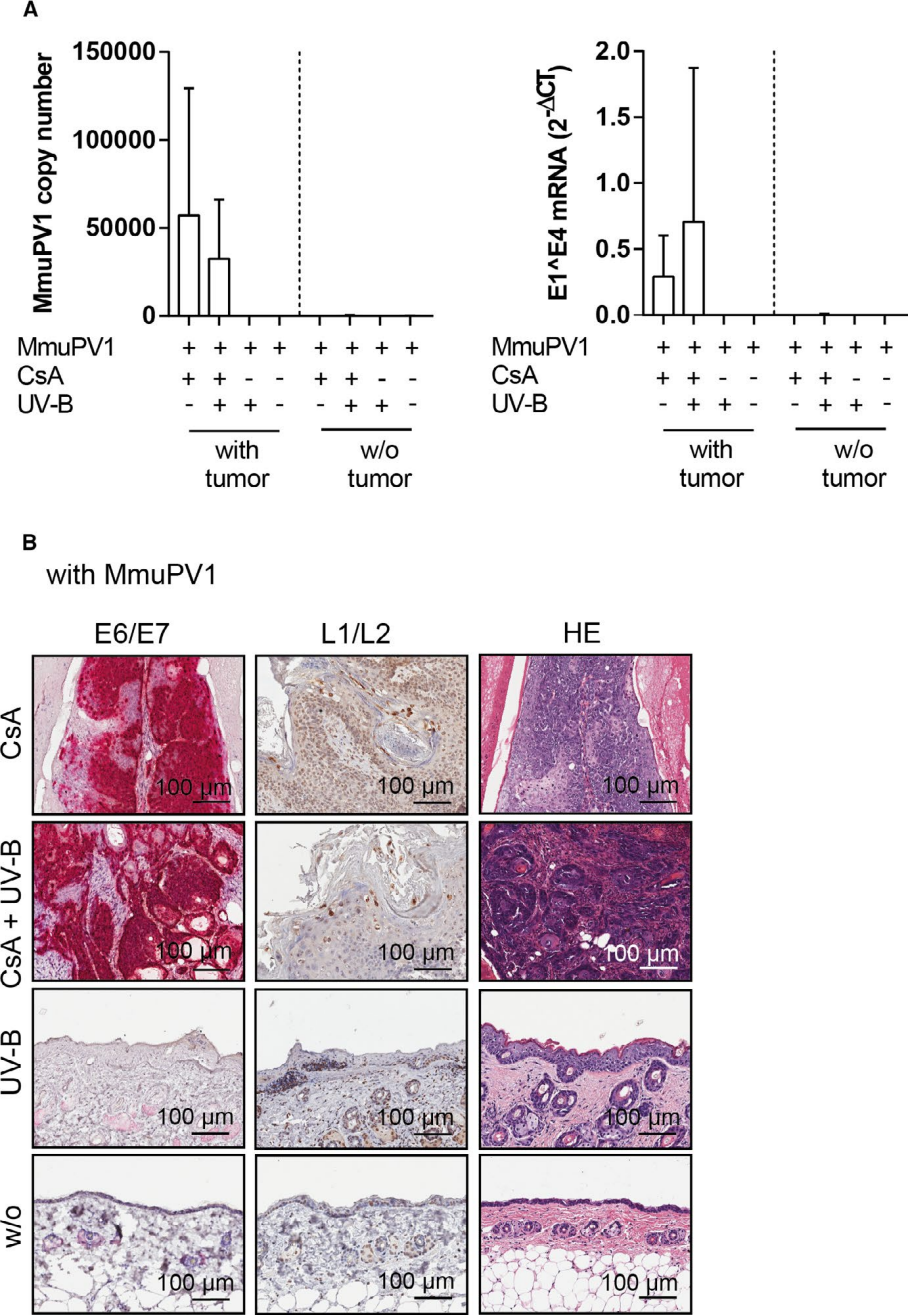
Viral presence in tumors on back skin. (A) Left panel: MmuPV1 genome copy numbers in back skin of MmuPV1‐infected mice stratified according to tumor development. Right panel: MmuPV1‐E1^E4 spliced transcripts in back skin tumors and nontumorous back skin of infected mice. (B) Left panel: MmuPV1‐E6/E7 mRNA in back skin of infected mice. Middle panel: IHC for MmuPV1‐L1/L2 capsid proteins in back skin. Right panel: corresponding HE‐stained sections

Detection of MmuPV1 genome and E1^E4 spliced transcripts was restricted to tumorous tissues of back (Figure 2A) and tail skin (data not shown), which had developed in virus‐infected, CsA‐treated, and CsA‐/UV‐B–treated mice at week 30 postinfection, but were not detected in nontumorous skin. Accordingly, the E6/E7 oncogene mRNA was abundantly present in the cSCCs and, to a lesser extent, in the premalignant tumors, but not in virus‐infected, nontumorous skin (Figure 2B, Figure S3A,B). Together with the presence of the L1/L2 capsid proteins in the uppermost layers of the tumors (Figure 2B), this revealed that MmuPV1 is transcriptionally active in the tumor entities and productive infection occurs. The viral markers were not detected in the tissues of uninfected mice and the results obtained in tail skin were equal to back skin.

### 3.3 MmuPV1 infection induces the expression of phosphorylated H2AX (γH2AX) in cSCCs

**FIGURE 3:**
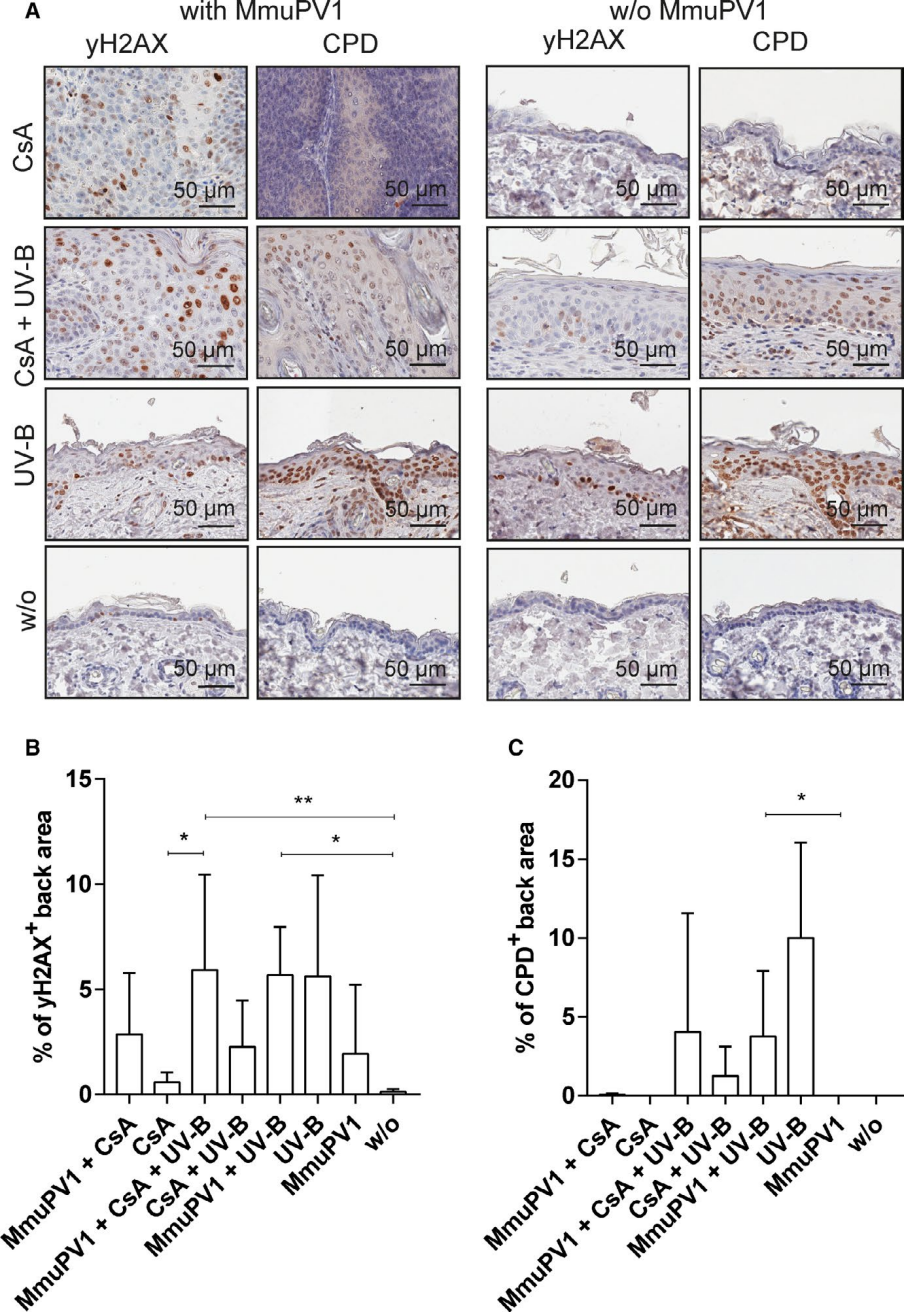
yH2AX and CPD staining of back skin. (A) Representative yH2AX (far‐left panel) and CPD (left panel) stainings of MmuPV1‐infected mice. Representative yH2AX (right panel) and CPD (far‐right panel) stainings of uninfected control mice. (B) Quantification of yH2AX‐immunopositive back skin area. (C) Quantification of CPD‐immunopositive back skin area

MmuPV1 infection induced γH2AX, a surrogate marker for DNA double‐strand breaks and chromatin instability, by 19‐fold in the tissues of back skin of non‐CsA/non‐UV‐B–treated and by 4.8‐fold in CsA‐treated mice compared to equally treated, uninfected controls (Figure 3A,B), indicating contribution of the viral oncogenes. Immunopositivity was restricted to tumorous tissues, whereas nontumorous skin areas were negative (Figure S4A). UV‐B irradiation generally induced low‐level expression of γH2AX; however, in virus‐infected mice, concomitant CsA administration allowed for 2.6‐fold elevated γH2AX expression compared to uninfected controls. Tumorous and nontumorous skin tissues stained positive for γH2AX, the latter presumably attributable to the effects of UV‐B (Figure S4A). Expression of γH2AX was not detected in the tissues of uninfected, non‐CsA/non‐UV‐B–treated and uninfected, CsA‐treated animals (Figure 3A,B).

Cyclobutane pyrimidine dimers (CPDs), which represent potentially mutagenic UV‐induced photoproducts, were only detectable in UV‐B–irradiated mice (Figure 3A,C). In the presence of CsA, MmuPV1 infection led to 3.1‐fold elevated, in the absence of CsA to 2.7‐fold reduced levels of CPDs compared to uninfected, equally UV‐B–treated littermates. CPDs were present in the keratinocytes of nontumorous skin, but not in the tumors (Figure S4B,C), indicating that inhibition of CPD repair by MmuPV1 might occur in CsA‐treated mice or that there is selection for cells with low CPD during tumorigenesis.

### 3.4 MmuPV1 does not degrade proapoptotic BAK

**FIGURE 4:**
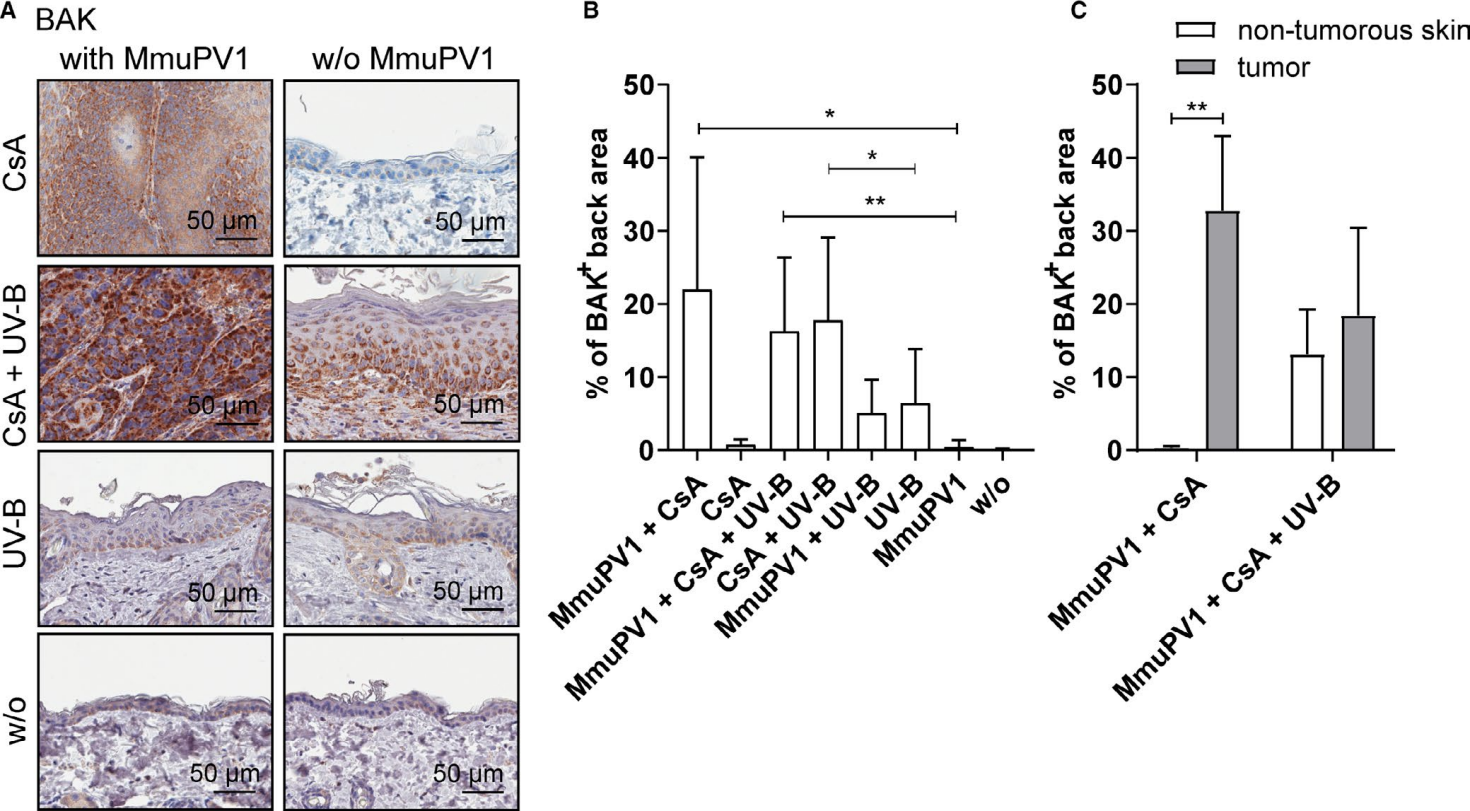
BAK staining of back skin. (A) Representative BAK stainings of MmuPV1‐infected (left panel) and uninfected (right panel) mice. (B) Quantification of BAK‐immunopositive back skin area. (C) Quantification of BAK immunostainings in tumorous and nontumorous back skin of MmuPV1‐infected, CsA‐treated and MmuPV1‐infected, CsA‐/UV‐B–treated mice

MmuPV1 infection elevated the levels of the proapoptotic effector protein BAK by 31.4‐fold in CsA‐treated mice compared to uninfected, CsA‐treated animals. Intense staining was observed in tumor tissues, but not in nontumorous skin, indicating increased apoptosis (Figure 4A‐C). Upon UV‐B exposure, BAK‐positive cells were readily detected throughout the skin tissues, demonstrating that BAK is involved in apoptosis signaling in response to UV‐B damage, but levels were not affected by MmuPV1 infection. In the presence of CsA, BAK levels were about 3‐fold higher after UV‐B irradiation, regardless of MmuPV1 infection. BAK was nearly undetectable in back skin of uninfected, non‐CsA/non‐UV‐B–treated and CsA‐treated mice (Figure 4A‐C).

### 3.5 MmuPV1‐induced cSCCs contain higher numbers of CD4^+^, CD8^+^, and FOXP3^+^, but not CD103^+^ T cells

**FIGURE 5:**
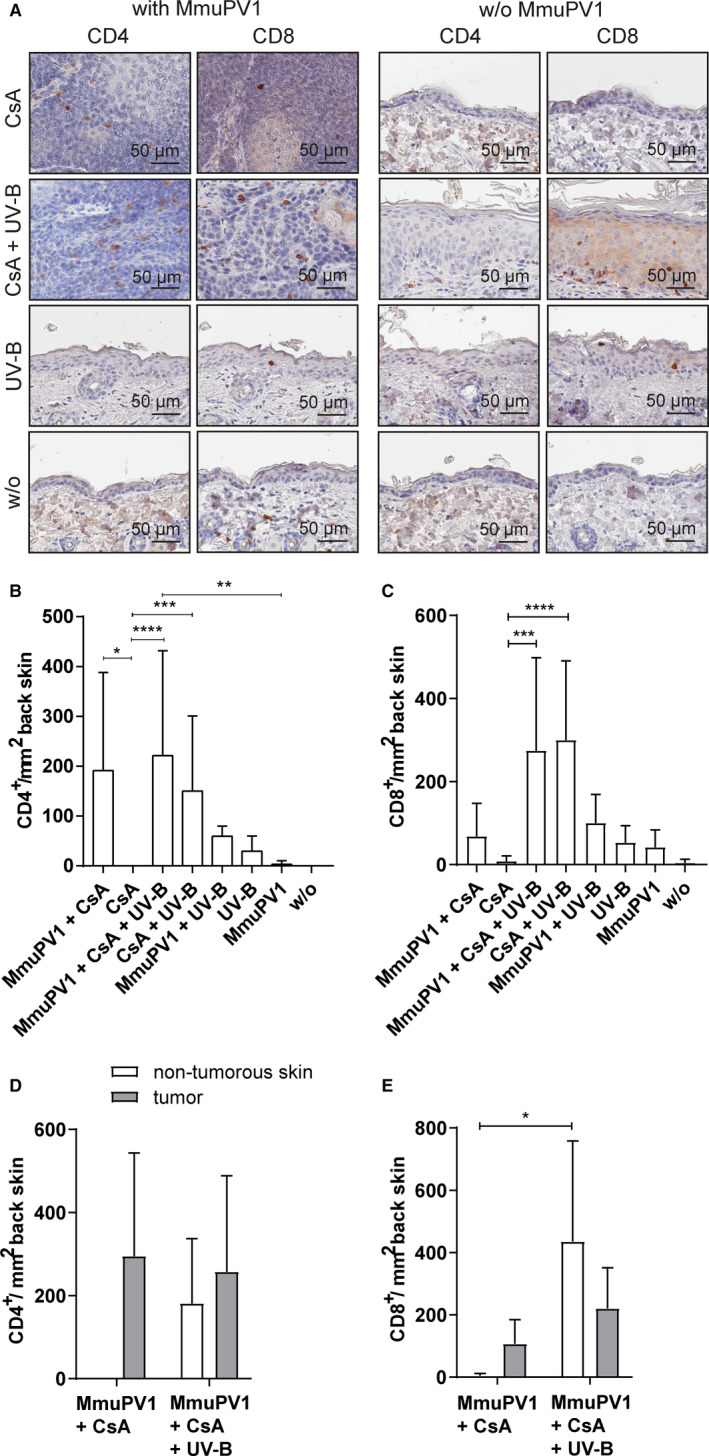
Presence of CD4^+^ and CD8^+^ T cells in back skin. (A) Representative CD4^+^ (far‐left panel) and CD8^+^ (left panel) stainings of MmuPV1‐infected mice. Representative CD4^+^ (right panel) and CD8^+^ (far‐right panel) stainings of uninfected control mice. (B) Quantification of CD4^+^‐immunopositive T cells per mm^2^ back skin in back skin. (C) Quantification of CD8^+^‐immunopositive T cells per mm^2^ back skin. (D) Quantification of CD4^+^‐immunopositive T cells in tumorous and nontumorous back skin of MmuPV1‐infected, CsA‐treated and MmuPV1‐infected, CsA‐/UV‐B–treated mice. (E) Quantification of CD8^+^‐immunopositive T cells in tumorous and nontumorous back skin of MmuPV1‐infected, CsA‐treated and MmuPV1‐infected, CsA‐/UV‐B–treated mice

MmuPV1 infection increased the numbers of CD4^+^ and CD8^+^ T cells in the skin tissues (Figure 5A‐C) throughout the experimental groups. Compared to uninfected, non‐CsA–/non‐UV‐B–treated animals, where CD4^+^ and CD8^+^ T cells were scarce, viral infection increased the mean CD4^+^ and CD8^+^ T cell numbers by 3.2‐ and 9.5‐fold, respectively. In CsA‐treated, but noninfected mice, CD4^+^ and CD8^+^ T cells remained undetectable. In contrast, after MmuPV1 infection of CsA‐treated mice, numerous CD4^+^ and CD8^+^ T cells were observed in the tissues showing a 191.9‐ and 8.1‐fold elevation of mean numbers, respectively, suggesting a promoting effect in the presence of CsA. T cell infiltration of the cSCCs, but not of nontumorous skin, was observed in virus‐infected mice (Figure 5D,E). In CsA‐/UV‐B–treated mice viral infection similarly increased the mean CD4^+^ T cell numbers, albeit not as pronounced, compared to uninfected, equally treated controls, with no effects seen in the CD8^+^ subset. However, analyses of T cell numbers in tumorous and nontumorous tissues revealed higher CD8^+^ T cell numbers in the nontumorous tissues of MmuPV1‐infected, CsA‐/UV‐B–treated mice than in the cSCCs of MmuPV1‐infected, CsA‐treated, and MmuPV1‐infected CsA‐/UV‐B–treated mice (Figure 5D,E).

Regulatory FOXP3^+^ T cells (Figure S5A,B) were generally less present in the skin tissues and not influenced by MmuPV1 infection, except for CsA‐treated animals. In these mice, infection led to 14.4‐fold higher mean numbers compared to uninfected controls. The cells were restricted to tumor tissues, but not found in nontumorous skin.

In addition, the numbers of tissue‐resident memory CD103^+^ T cells (Figure S5A,C) were increased in skin tissues of virus‐infected compared to uninfected mice, in particular in the absence of CsA or UV‐B (6.5‐fold). In the presence of CsA, the increase was not as pronounced (1.2‐fold); however, fewer tumor‐infiltrating CD103^+^ lymphocytes were observed in the cSCCs of virus‐infected mice compared to nontumorous skin.

### 3.6 MmuPV1 infection modestly increases CD4^+^, CD8^+^, and CD45R^+^ in the draining lymph nodes

**FIGURE 6:**
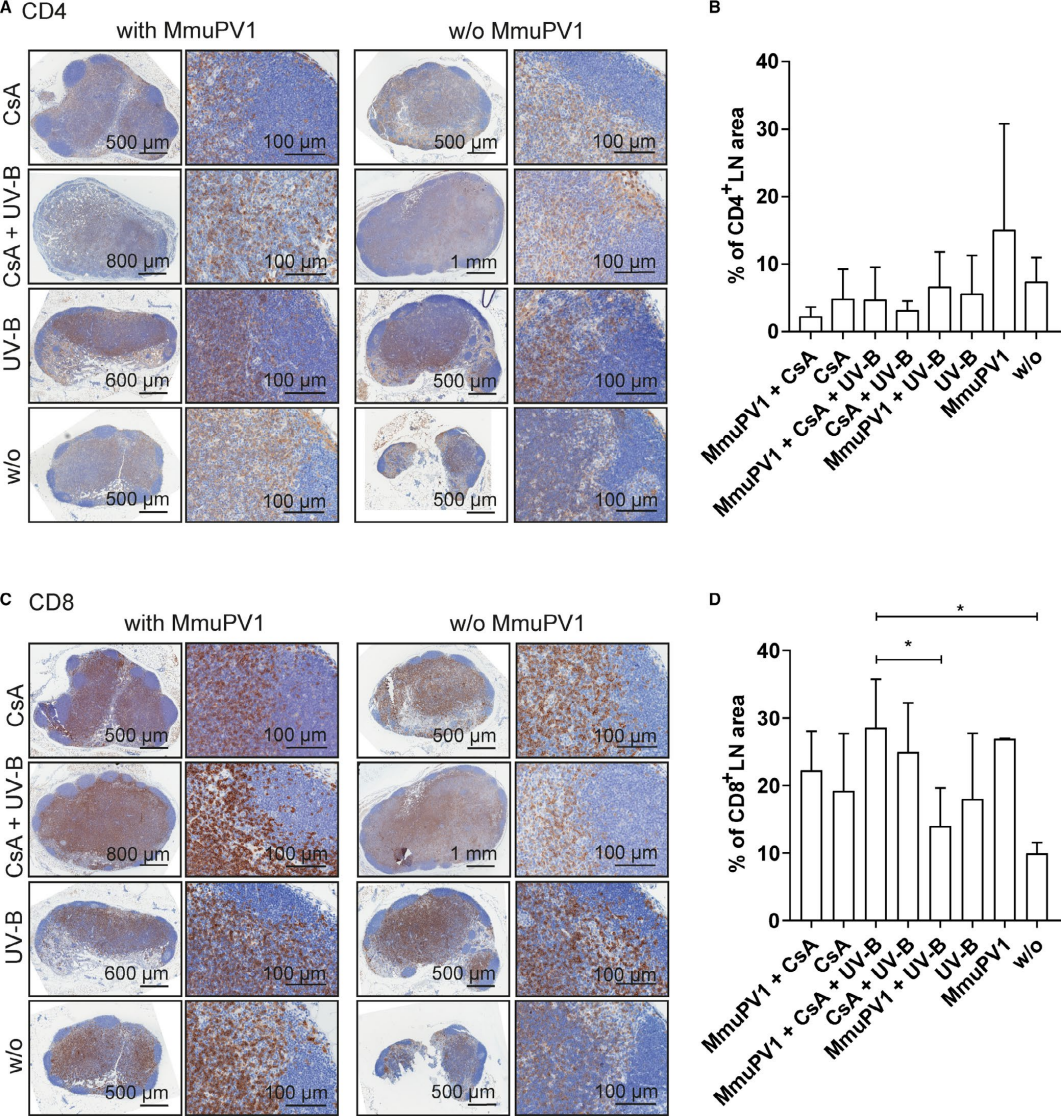
Presence of CD4^+^ and CD8^+^ T cells in draining lymph nodes. (A) CD4^+^ stainings of lymph nodes derived from MmuPV1‐infected mice (far‐left and left panels). CD4^+^ stainings of lymph nodes derived from uninfected mice (far‐right and right panels). Overview and higher magnification of the same tumors are shown. (B) Quantification of CD4^+^‐immunopositive lymph node areas. (C) CD8^+^ stainings of lymph nodes derived from MmuPV1‐infected mice (far‐left and left panels). CD8^+^ stainings of lymph nodes derived from uninfected mice (far‐right and right panels). Overview and higher magnification of the same tumors are shown. (D) Quantification of CD8^+^‐immunopositive lymph node areas

Similar to skin, MmuPV1‐infected mice had generally larger CD4^+^‐ (Figure 6A,B) and CD8^+^‐ (Figure 6C,D) immunopositive areas in the draining lymph nodes than uninfected controls, although the effects were modest (up to 2.1‐ and 2.7‐fold in non‐CsA/non‐UV‐B–treated animals, respectively). Systemic CsA administration reduced the CD4^+^‐immunopositive areas in infected compared to uninfected mice by 2.2‐fold, while the CD8^+^‐positive areas remained unaltered.

**FIGURE 7:**
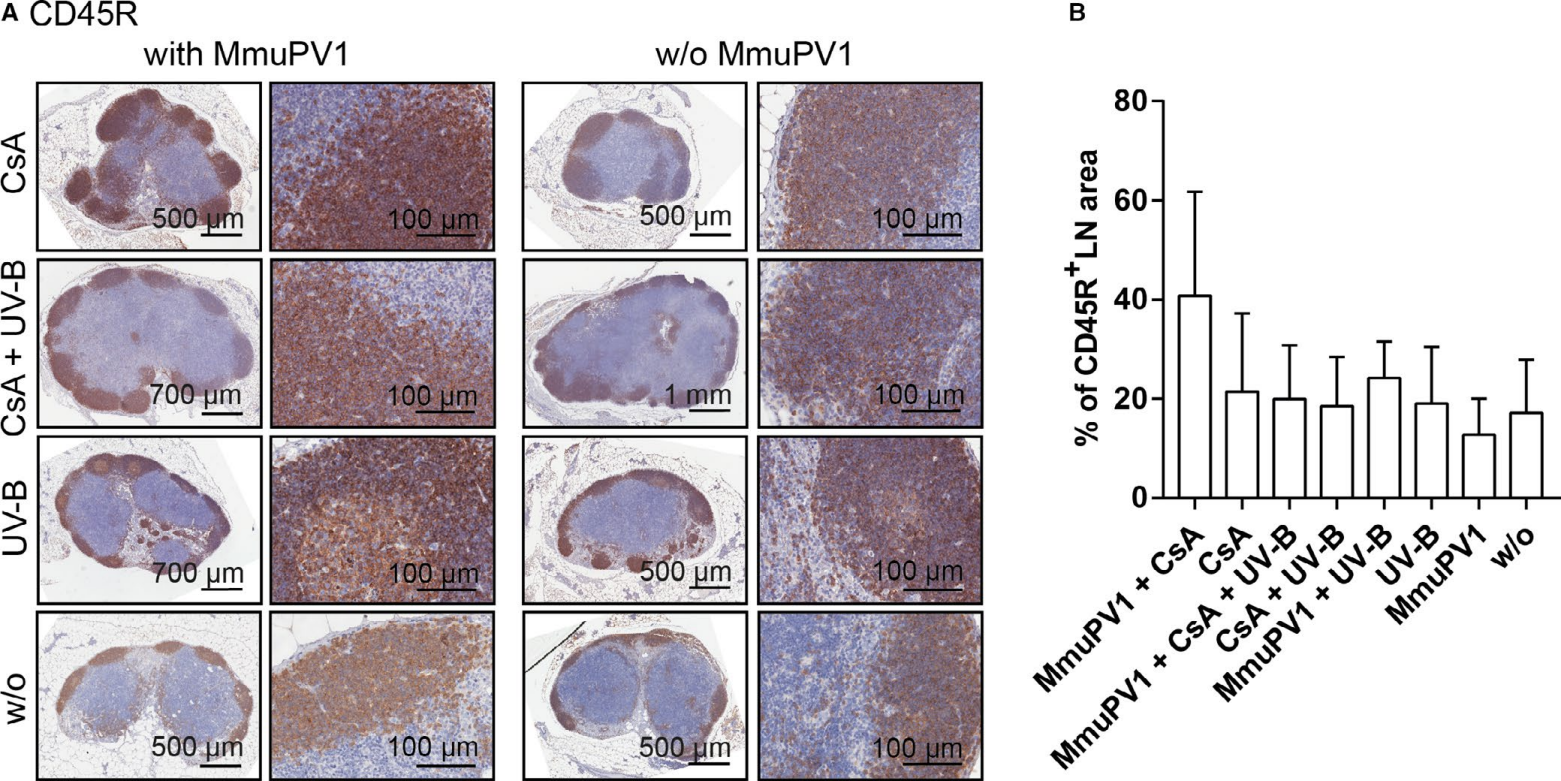
Presence of CD45R^+^ T cells in draining lymph nodes. (A) CD45R^+^ stainings of lymph nodes derived from MmuPV1‐infected mice (far‐left and left panels). CD45R^+^ stainings of lymph nodes derived from uninfected mice (far‐right and right panels). Overview and higher magnification of the same tumors are shown. (B) Quantification of CD45R^+^‐immunopositive lymph node areas

CD45R^+^‐immunopositive (Figure 7A,B) areas were similarly modestly elevated in MmuPV1‐infected mice compared to the appropriate uninfected controls, in particular in CsA‐treated animals (1.4‐ and 1.9‐fold, respectively). A similar pattern was observed in mice, which had been MmuPV1 infected, and uninfected controls.

### 3.7 CsA treatment impairs the production of functionally active, MmuPV1‐specific neutralizing antibodies

**FIGURE 8:**
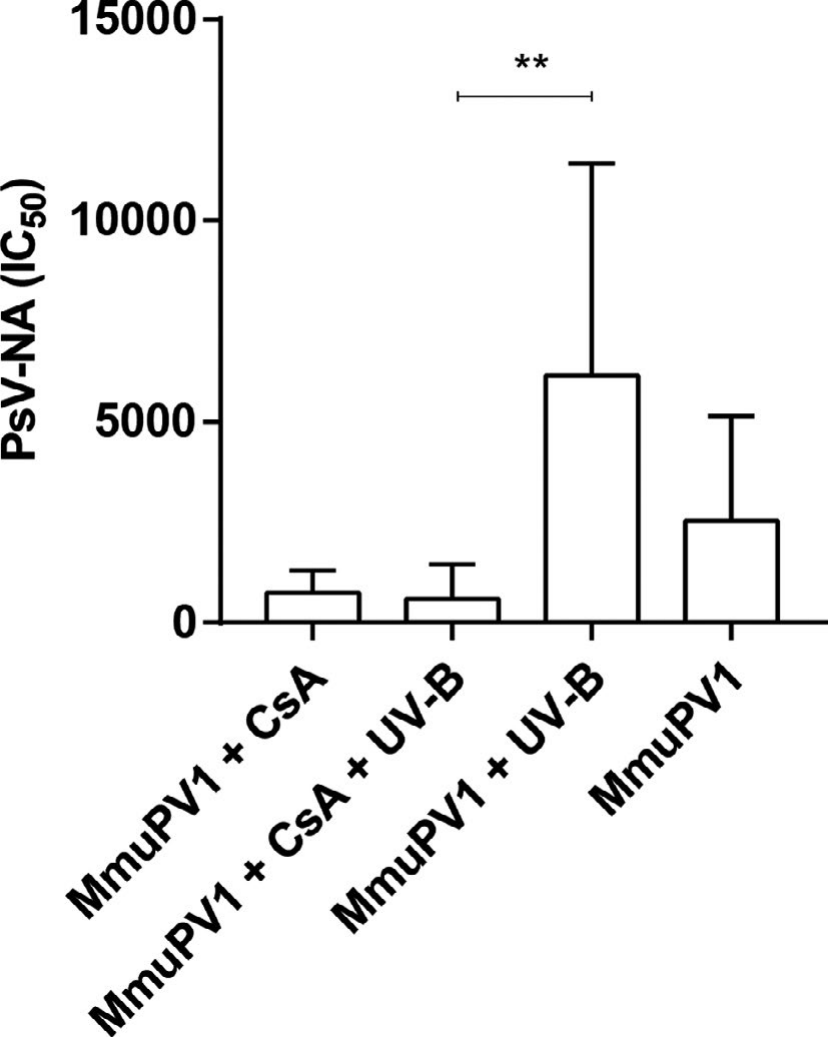
MmuPV1‐specific neutralizing antibodies in mouse sera. IC_50_ titers were determined by PsV‐NA

All MmuPV1‐infected mice, irrespective of the immune state, generated antibodies against the viral L1/L2 capsid proteins at variable levels, as determined by particle‐ELISA (Figure S6) and PsV‐NA (Figure 8). On the contrary, neutralizing antibodies which represent the antibody class capable of preventing papillomaviral (re‐)infection^26^ were reduced by 3.4‐ and 4.3‐fold in CsA‐treated and CsA‐/UV‐B–treated mice. Both assays, particle‐ELISA and PsV‐NA, correlated well (*r* = .7754; 95% CI: 0.5962‐0.8810). Uninfected animals lacked seroresponses against MmuPV1 capsid (data not shown).

### 3.8 MmuPV1‐induced cSCC cells are tumorigenic in vivo independently of viral oncogene expression

**FIGURE 9:**
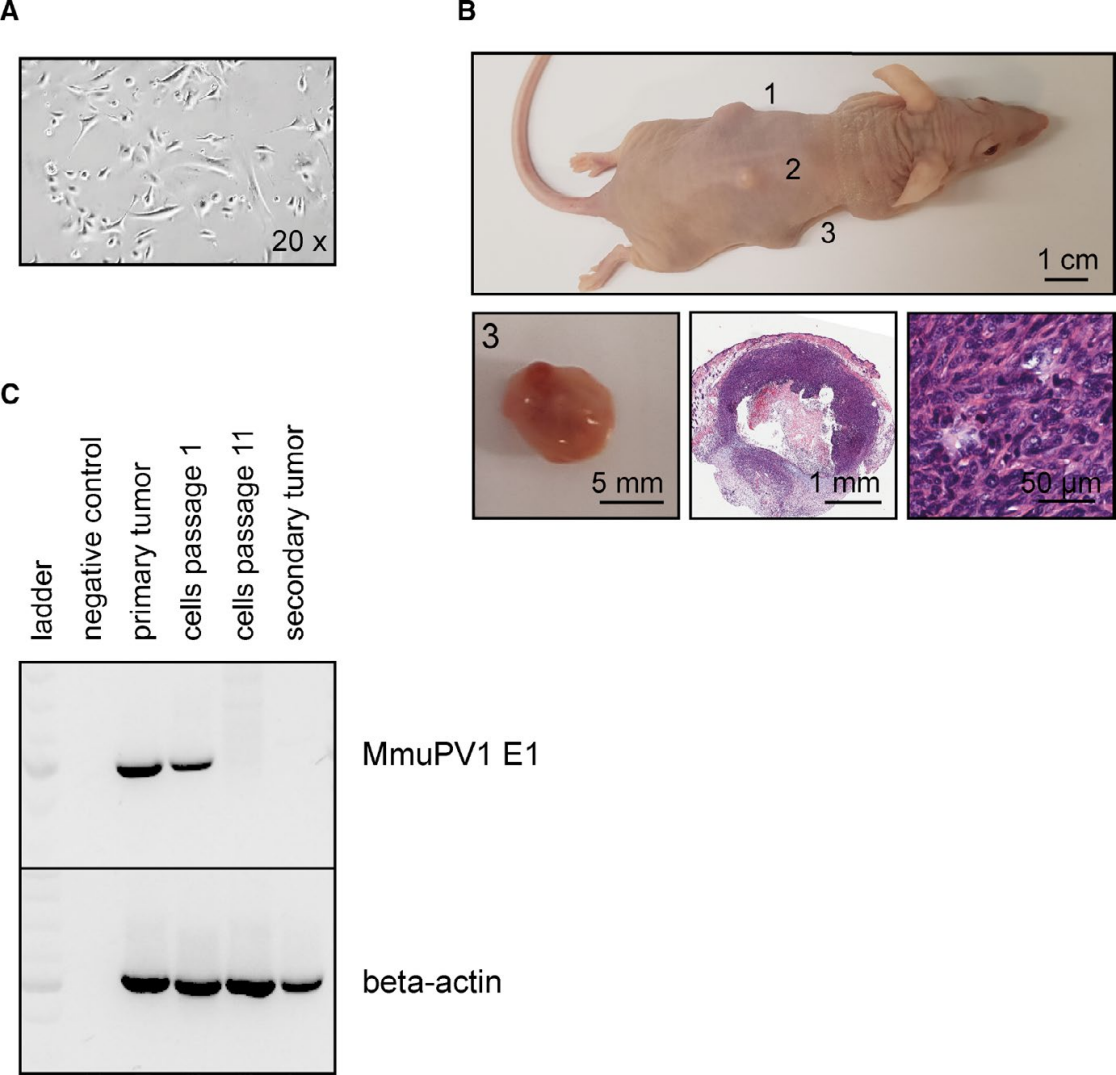
Tumor cell explant and virus‐independent secondary tumor formation. (A) Primary tumor cells established from a MmuPV1‐infected, CsA‐/UV‐B–treated mouse. (B) Intradermal administration of primary cSCC cells of passage 11 to NMRI‐Foxn1^nu/nu^ mice gave rise to secondary tumors. One representative NMRI‐Foxn1^nu/nu^ mouse 30 days postinjection. Secondary tumors after procurement. Overview and higher magnification of a HE‐stained secondary cSCC. (C) Presence of MmuPV1 DNA in the primary cSCC and in the early passages of the primary cSCC cell line. Absence of MmuPV1 DNA in late passages of the primary cSCC cell line and in the secondary tumor

Primary tumor cells established from a cSCC readily gave rise to malignant tumors after administration to NMRI‐Foxn1^nu/nu^ mice (Figure 9A,B). Both, parental and secondary tumors stained positive for pan‐CK and CD34; however, vimentin staining was more abundant and homogeneous in the secondary cSCCs (Figure S7). The MmuPV1 E1 gene was readily detectable in the primary tumor and in the primary cells’early passages, but was lost at passage 11 and in the secondary tumors derived from passage 11 cells (Figure 9C). Nevertheless, secondary cSCCs formed in the absence of viral DNA, demonstrating that maintenance of the malignant phenotype of the cell line as well as outgrowth of the secondary tumors was virus independent.

## 4 DISCUSSION

The role of cutaneous genus beta‐HPVs in the pathogenesis of keratinocyte carcinomas of the skin in the normal immunocompetent population has long been a matter of debate. In OTRs, whose viral infections are not adequately controlled due to iatrogenic immunosuppression, the highly elevated risk to develop cSCCs raises the possibility of a viral contribution to pathogenesis. The mouse model presented herein demonstrated several important findings: (1) the unequivocal necessity of MmuPV1 in skin cancer formation (as in the absence of viral infection malignancies did not develop); (2) the importance of an intact immune system, as iatrogenic immunosuppression by CsA was a prerequisite for skin cancer development; (3) the dispensability of UV‐B in MmuPV1‐induced skin carcinogenesis, as cSCCs arose in the absence of UV‐B; (4) corroboration of the hit‐and‐run mechanism proposed for papillomavirus‐induced skin carcinogenesis.

To establish this MmuPV1‐induced skin cancer model, the inbred FVB/NCrl strain was chosen based on its elevated susceptibility to MmuPV1 infection and high sensitivity to chemical carcinogen‐induced skin cancers.^27^, ^28^ MmuPV1 infects murine skin of several mouse strains, although disease development is strain dependent and largely restricted to immunocompromised, particularly T cell deficient, animals.^29^ In our experimental setting, MmuPV1 infection in the presence of immunosuppressive CsA, which is widely used in the prevention and treatment of solid organ transplant rejection, allowed for consistent and robust cSCC development in the vast majority of mice, whereas in the absence of CsA, tumor outgrowth was not observed. This is presumably due to timely clearance of viral infection, indicated by the lack of detectable virus 30 weeks after infection, by an intact cellular murine immune system in the absence of CsA, thus hampering viral persistence and subsequent malignant conversion. The presence of CsA seems to allow for uncontrolled viral replication and productive infection, which in turn can initiate the first steps in carcinogenesis, for example, by the induction of γH2AX. In addition, CsA inhibits the production of cytokines involved in T cell activation and UV‐B–induced apoptosis and DNA repair in keratinocytes.^30^, ^31^, ^32^, ^33^ Hence, CsA can contribute to cancer formation not only by suppressing the antiviral and/or antitumor immunity, but also by impairing the repair of mutated DNA. Further investigations of the impact of other immunosuppressants employed in OTRs, such as the calcineurin inhibitor tacrolimus or the mTOR inhibitor rapamycin, will be performed in this mouse model, aiming at dissecting possible differences in their carcinogenic effects.

MmuPV1 infection recruited various T cell populations to the site of infection, regardless of CsA, indicating epithelial trafficking of T cells was not impaired, and the cellular infiltrate was predominantly found in the cSCCs. Previously, both CD4^+^ and CD8^+^ T cells have been individually shown to protect against MmuPV1 infection in C57BL/6, but not in SENCAR mice.^27^ The situation in the FVB/NCrl strain, however, is not clear. Regardless of the effects of T cells on controlling viral infection, a differential function of CD4^+^ and CD8^+^ T cells in skin cancer development was reported employing the classical murine model for two‐stage chemical cutaneous carcinogenesis.^34^, ^35^, ^36^ However, the specific T cell population, which exerts protection against skin carcinogenesis is not completely identified. Yusuf et al. showed that CD4^+^‐deficient mice had a lower tumor burden than CD8^+^‐deficient mice and vice versa, concluding that CD8^+^ T cells protect from skin cancer development, whereas CD4^+^ T cells have a promotive effect.^34^ On the contrary, in mice on a FVB/N background cancer‐promoting T cells were allocated to the T cell receptor (TCR) αβ^+^CD8^+^ compartment.^35^, ^36^, ^37^ This specific population was thought to augment malignant progression through favoring a proinflammatory tumor environment and inhibiting CD8^+^ cytolytic responses. The contribution of T cells to MmuPV1‐induced tumor development in FVB/NCrl mice may be a mixture of competing and coexisting forces. We have not yet attempted to unravel the specific subset responsible for protecting against and/or promoting skin cancer development in our cancer‐bearing FVB/NCrl mice, and we will also investigate whether CsA can impair T cell function to clear MmuPV1 infection. After MmuPV1 infection, reduced anti‐MmuPV1–neutralizing antibody titers (assessed by PsV‐NA) were observed in CsA‐treated animals, although anti‐MmuPV1‐L1/L2 antibodies (assessed by particle‐ELISA) were readily detectable. This indicates that CsA could impair T cell–mediated B cell maturation and function. In the draining lymph nodes of infected and uninfected, CsA‐treated mice, CD4^+^, CD8^+^, and CD45R^+^ cells were not significantly reduced; however, germinal centers, the sites essential for B cell activation, proliferation, and production of high‐affinity antibodies,^38^ seemed to be absent. Neutralizing antibodies can prevent viral infection; however, antibodies are generated after the establishment of infection in the target cell and do not promote clearance of infection. Hence, it is unlikely that the lack of or the reduced levels of antibodies contributed to the prevention of virally induced tumor formation in our mice. In this line, cSCC development did not correlate with the levels of neutralizing antibodies (Figure S6C,D).

Interestingly, UV‐B, the main causative factor in the pathogenesis of skin cancer, was not necessary for cSCC formation in our model system. MmuP1‐infected, UV‐B–irradiated mice did not develop malignant tumors, except for one mouse which had one cSCC on the tail. Similarly, in another report UV‐B irradiation of MmuPV1‐infected mice did not allow for cSCC outgrowth on tail, but on ear skin.^18^ We have not investigated cSCC development in ear skin due to the vulnerability of skin at this localization, but have restricted to investigations of dorsal and tail skin. Differences in the keratin networks, transcriptional factors, and MHC expression between individual anatomical parts on the mouse were reported,^29^, ^39^ that could contribute to site‐specific susceptibility to tumorigenesis (skin of ear versus back and tail skin).^18^, ^29^ Intriguingly, cSCCs on back skin were 1.7 times more frequent in CsA‐treated compared to CsA‐/UV‐B–treated mice. Possible explanations are that in the latter group efficiency of infection was hampered by UV‐B irradiation, which had already induced a certain degree of skin hardening prior to inoculation. We observed an increased thickening of the epidermis, which was most pronounced in mice of the CsA‐/UV‐B– followed by the CsA treatment group (data not shown), presumably due to different factors, such as the inoculation process, UV‐B irradiation and as side effect of CsA, which could interfere with lesional outgrowth. Furthermore, the local T cell infiltrate present in the murine skin could have influenced the outcome. The higher numbers of CD8^+^ T cells present in the nontumorous skin of MmuPV1‐infected, CsA‐/UV‐B–treated mice induced by UV‐B irradiation could exert a certain degree of control in carcinogenesis, such as prevention of tumor outgrowth. In the cSCCs of MmuPV1‐infected, CsA‐/UV‐B–treated mice or in tissues of MmuPV1‐infected, CsA‐, but not UV‐B–treated mice, the low numbers of CD8^+^ T cells, however, may not suffice to inhibit tumor growth. Similarly, the higher numbers of CD8^+^ T cells induced by UV‐B in the cSCCs of the MmuPV1‐infected, CsA‐/UV‐B–treatment group compared to MmuPV1‐infected, CsA‐/non‐UV‐B–treated mice could account for the prevention of uncontrolled tumor expansion, thus, resulting in reduced tumor size. In the absence of an additional tumor‐promoting effect caused by CsA (e.g., in the MmuPV1‐infected, UV‐B–treatment group), the capability of the T cells, albeit lower in absolute numbers, may be sufficient to efficiently prevent cancer development. The effect seems to be a local rather than a systemic phenomenon, as T cell numbers were more or less equal in the draining lymph nodes of MmuPV1‐infected, CsA‐treated and MmuPV1‐infected, CsA‐/UV‐B–treated mice. In addition, keratinocytes respond to low‐intensity UV irradiation by increasing DNA repair and inducing expression of the tumor suppressor protein p16, hence counteracting the process of virally induced malignant transformation.^40^ In contrast to a recently published MmuPV1 mouse model,^18^ herein mice were exposed to cumulative rather than a single dose of UV‐B, recapitulating the situation in humans, where lifelong sun exposure is the major causal factor in the development of keratinocyte skin malignancies. However, we showed that while the applied UV‐B dose induced DNA damage in the irradiated tissues of mice, as demonstrated by the expression of γH2AX, a surrogate marker for DNA double‐strand breaks and chromatin instability, interestingly, MmuPV1 per se seems to be capable to induce genomic instability, even without concomitant irradiation. Inhibition of CPD repair by MmuPV1 might occur in the presence of CsA, which allows for elevated viral replication in the skin tissues, possibly favoring carcinogenesis. Another important mechanism in skin carcinogenesis is the inhibition of keratinocyte apoptosis in response to UV damage, mediated by the viral E6 oncoproteins. In this line, the E6 of several beta‐HPVs were shown to inactivate BAK^41^, ^42^ and the survival of DNA‐damaged cells promotes the progression of skin malignancies. Conversely, to beta‐HPVs, we did not observe BAK degradation in skin tissues of infected mice. This could be due to differences in the susceptibility to UV‐B light between mouse and human keratinocytes because of their evolutional history. It could be possible that neither the intrinsic antiapoptotic mechanisms in the keratinocytes nor those provided by MmuPV1 infection are enough to fully overcome the UV‐B–induced kill of the mouse keratinocytes, whether normal or transformed. Since mice are evolutionarily not a subject to UV damage as humans, it is possible that MmuPV1 may not have evolved ways of counteracting it to the extent that skin‐tropic HPVs have.

In summary, our data provide evidence that MmuPV1 has carcinogenic potential and skin infection causes cSCCs in mice upon systemic immunosuppression even in the absence of UV‐B light. Strikingly, the “hit‐and‐run” mechanism proposed for beta‐HPVs applies to the MmuPV1‐induced skin cancer in an extended sense, as we demonstrated that our primary tumor cell line originating from a MmuPV1‐induced cSCCs became immortalized, lost viral DNA during passaging and was able to form de novo tumors in vivo independently of viral gene expression. The abundance of vimentin immunopositivity in our secondary cSCCs might be indicative of epithelial‐to‐mesenchymal transition. In this scenario, the virus‐infected keratinocytes become malignant and gradually assume a mesenchymal cell phenotype paralleled by acquisition of vimentin. This allows for enhanced migratory capacity and altered responsiveness to apoptotic stimuli, facilitating cancer invasion and progression with metastatic expansion, even when the virus gets lost. Hence, this study may instigate investigations regarding different options to prevent development or restrict (metastatic) invasion of cSCCs in OTRs, such as antiviral strategies (e.g., vaccination prior to organ transplantation, timely antiviral treatment), replacement of CsA by other immunosuppressants with less cancer‐ and/or virus‐promoting properties, and strategies that inhibit and/or reverse epithelial‐to‐mesenchymal transition.

## DISCLOSURE

The authors of this manuscript have no conflicts of interest to disclose as described by the *American Journal of Transplantation*.

## Supporting information


**Figure 1:** Experimental set‐up. Immunocompetent FVB/NCrl mice were infected with 1x10^10^ MmuPV1 virions per site on the back and tail skin on day 0. CsA treatment and UV‐B irradiation were started one week prior to infection. CsA was administered subcutaneously at a dose of 75 mg/kg body weight 5 times per week for the first eleven weeks and subsequently 3 times per week until end of experiment in week 30 post‐infection. UV‐B irradiation was performed 3 times per week with a starting dose of 120 mJ/cm^2^. The UV‐B dose was increased weekly until the final dose of 450 mJ/cm^2^ was reached in week 20 post‐infection. Irradiation was continued with the final dose until week 30 post infection.

**Figure 2:** Tumor incidence after experimental MmuPV1 skin infection on tail skin. A) Tumor incidence on tail skin in MmuPV1‐infected mice at week 30 post‐infection. Uninfected mice did not develop skin tumors. B) Time course of tumor outgrowth on tail skin. Tumor length is given in mm. C) Representative mouse of each experimental group with corresponding HE image. Left panel: MmuPV1‐infected, right panel: uninfected mice.

**Figure 3:** Viral presence in tumors on back skin. A) Left panel: lower magnification of E6/E7 mRNA present in representative cSCCs of the back. Right panel: corresponding HE stainings. B) Absence of E6/E7 mRNA in infected, adjacent non‐tumorous back skin tissues.

**Figure 4:** Quantification of yH2AX and CPD staining of back skin. A) Quantification of yH2AX‐immunopositivity in tumorous and non‐tumorous skin of MmuPV1‐infected, CsA‐treated and MmuPV1‐infected, CsA‐/UV‐B‐treated mice. B) Representative IHC staining for CPD of tumorous and non‐tumorous skin. C) Quantification of CPD‐immunopositivity in tumorous and non‐tumorous skin of MmuPV1‐infected, CsA‐treated and MmuPV1‐infected, CsA‐/UV‐B‐treated mice.

**Figure 5:** FOXP3^+^ and CD103^+^ T‐cells in back skin. A) Representative FOXP3^+^ (far left panel) and CD103^+^ (left panel) stainings of MmuPV1‐infected mice. Representative FOXP3^+^ (right panel) and CD103+ (far right panel) stainings of uninfected control mice. B) Quantification of FOXP3^+^‐immunopositive T‐cells in back skin; immunopositive T‐cells are given in numbers per mm^2^ back skin. C) Quantification of CD103^+^‐immunopositive T‐cells in back skin; immunopositive T‐cells are given in numbers per mm^2^ back skin. D) Quantification of FOXP3^+^‐immunopositive T‐cells in tumorous and non‐tumorous skin of MmuPV1‐infected, CsA‐treated and MmuPV1‐infected, CsA‐/UV‐B‐treated mice. E) Quantification of CD103^+^‐immunopositive T‐cells in tumorous and non‐tumorous skin of MmuPV1‐infected, CsA‐treated and MmuPV1‐infected, CsA‐/UV‐B‐treated mice.

**Figure 6:** MmuPV1‐specific antibodies in mouse sera. A) MmuPV1‐specific antibodies were determined by particle‐ELISA. B) Correlation of MmuPV1‐specific antibodies with neutralizing antibodies. C) Correlation of MmuPV1‐neutralizing antibodies with back tumor area D) Correlation of MmuPV1‐neutralizing antibodies with tail tumor length.

**Figure 7:** Comparison of pan‐cytokeratin, vimentin and CD34‐staining of primary and secondary cSCCs. Left side: cSCC induced on back skin by MmuPV1 infection in a CsA‐/UV‐B‐treated mouse. Right side: Secondary cSCC which had developed after administration of primary cSCC cells into a NMRIFoxn1^nu/nu^ mouse. The corresponding HE staining of the cSCCs is depicted in the first row.

## Data Availability

The data that support the findings of this study are available from the corresponding author upon reasonable request.
