# Supplementary figures and images for: Mus musculus papillomavirus 1 is a key driver of skin cancer development upon immunosuppression
Source: Am J Transplant. 2020 Nov 3;21(2):525–39. doi: 10.1111/ajt.16358 (PMC7894140; doi:10.1111/ajt.16358)

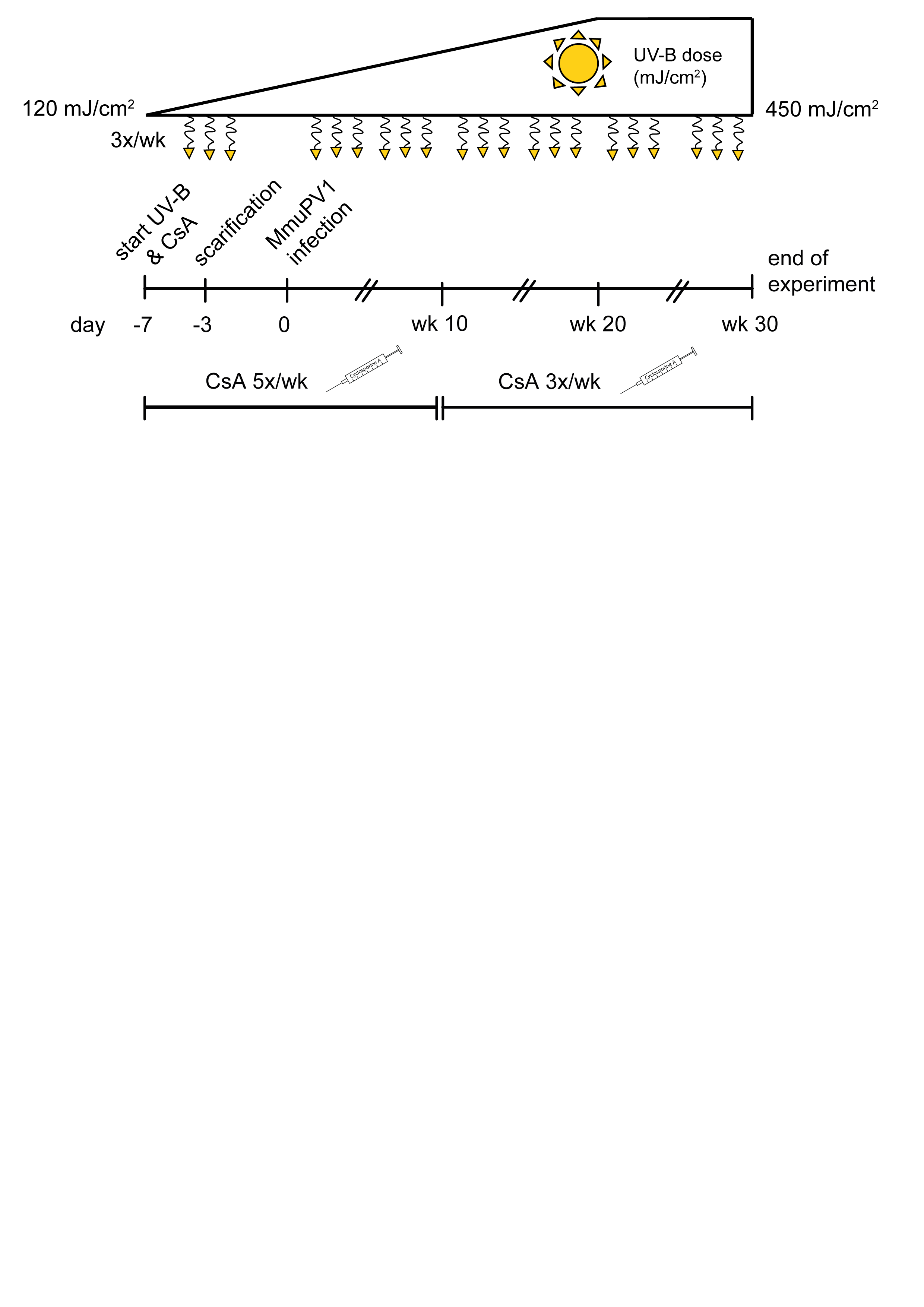

Supplement: Supplementary file 1 — Figure 1: Experimental set‐up. Immunocompetent FVB/NCrl mice were infected with 1x10^10^ MmuPV1 virions per site on the back and tail skin on day 0. CsA treatment and UV‐B irradiation were started one week prior to infection. CsA was administered subcutaneously at a dose of 75 mg/kg body weight 5 times per week for the first eleven weeks and subsequently 3 times per week until end of experiment in week 30 post‐infection. UV‐B irradiation was performed 3 times per week with a starting dose of 120 mJ/cm2. The UV‐B dose was increased weekly until the final dose of 450 mJ/cm2 was reached in week 20 post‐infection. Irradiation was continued with the final dose until week 30 post infection. [file AJT-21-525-s001.tif]

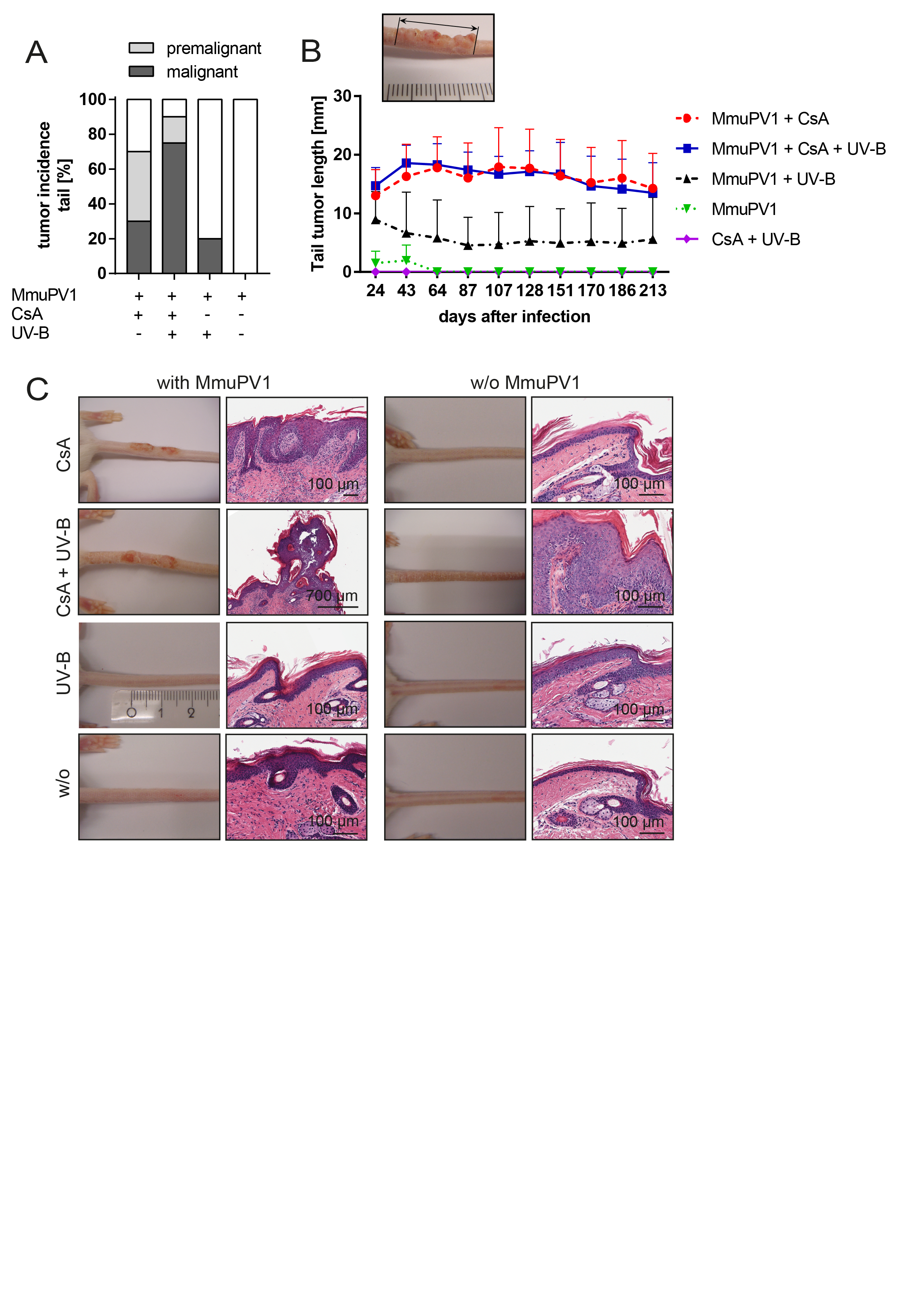

Supplement: Supplementary file 2 — Figure 2: Tumor incidence after experimental MmuPV1 skin infection on tail skin. A) Tumor incidence on tail skin in MmuPV1‐infected mice at week 30 post‐infection. Uninfected mice did not develop skin tumors. B) Time course of tumor outgrowth on tail skin. Tumor length is given in mm. C) Representative mouse of each experimental group with corresponding HE image. Left panel: MmuPV1‐infected, right panel: uninfected mice. [file AJT-21-525-s002.tif]

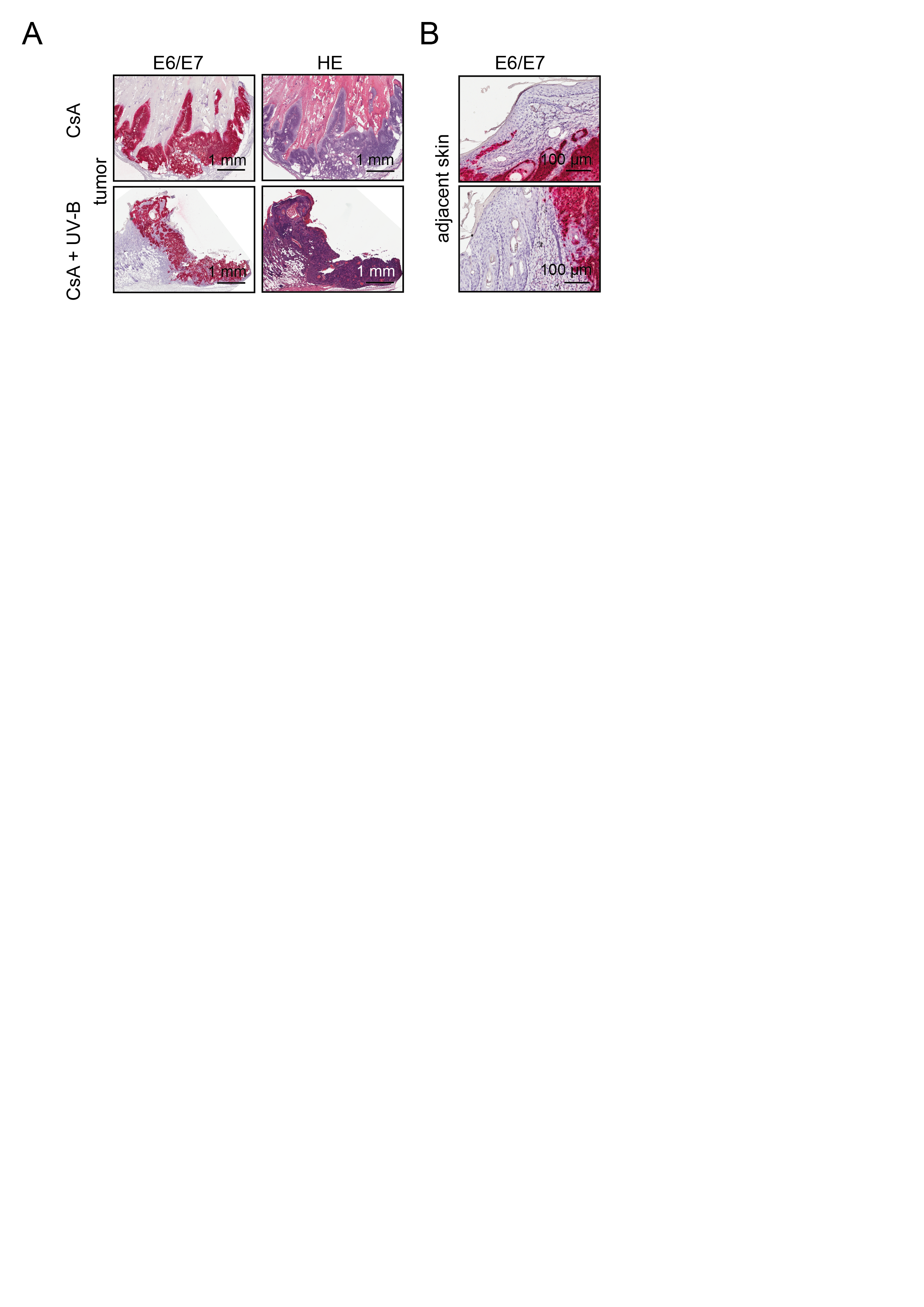

Supplement: Supplementary file 3 — Figure 3: Viral presence in tumors on back skin. A) Left panel: lower magnification of E6/E7 mRNA present in representative cSCCs of the back. Right panel: corresponding HE stainings. B) Absence of E6/E7 mRNA in infected, adjacent non‐tumorous back skin tissues. [file AJT-21-525-s003.tif]

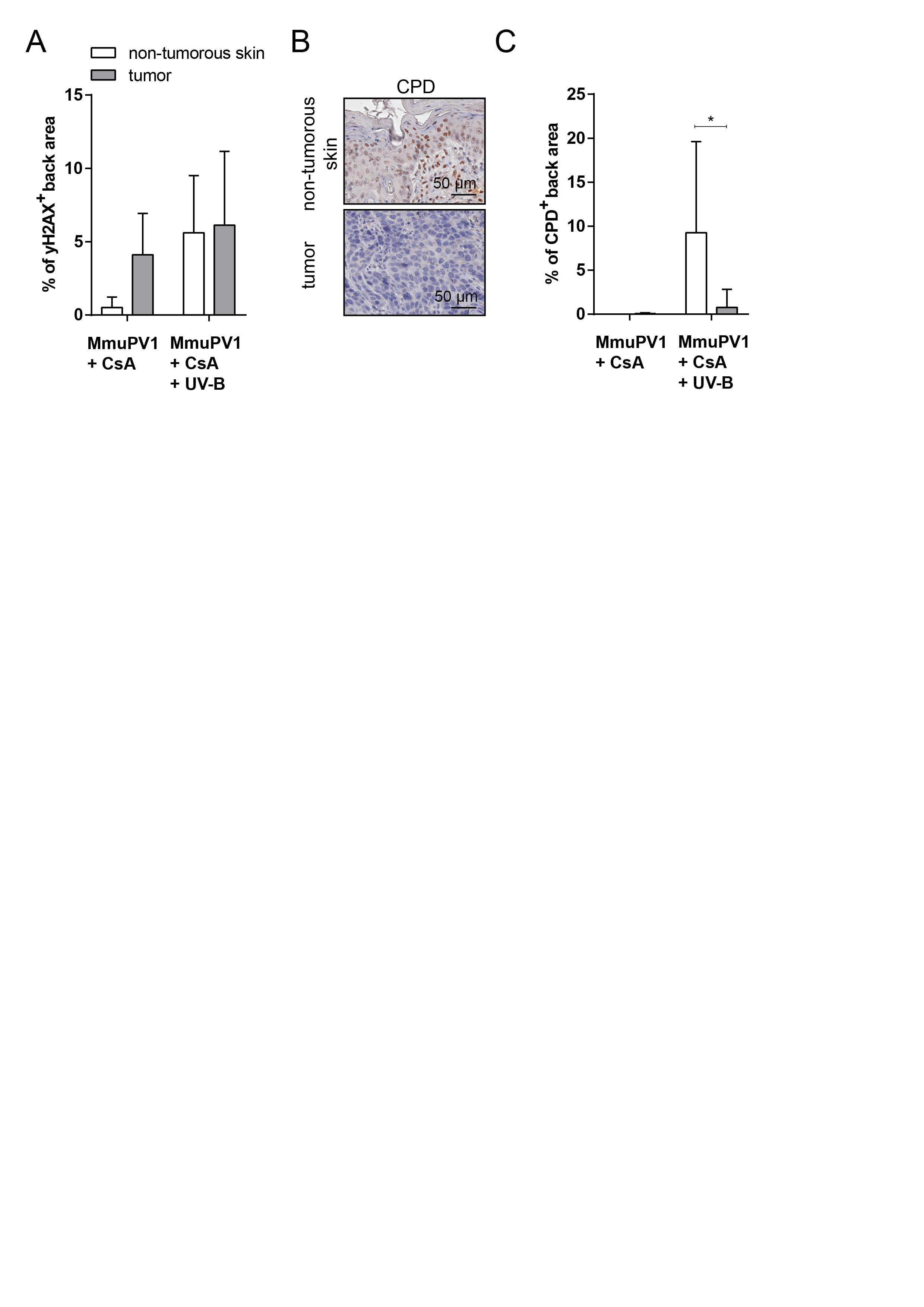

Supplement: Supplementary file 4 — Figure 4: Quantification of yH2AX and CPD staining of back skin. A) Quantification of yH2AX‐immunopositivity in tumorous and non‐tumorous skin of MmuPV1‐infected, CsA‐treated and MmuPV1‐infected, CsA‐/UV‐B‐treated mice. B) Representative IHC staining for CPD of tumorous and non‐tumorous skin. C) Quantification of CPD‐immunopositivity in tumorous and non‐tumorous skin of MmuPV1‐infected, CsA‐treated and MmuPV1‐infected, CsA‐/UV‐B‐treated mice. [file AJT-21-525-s004.tif]

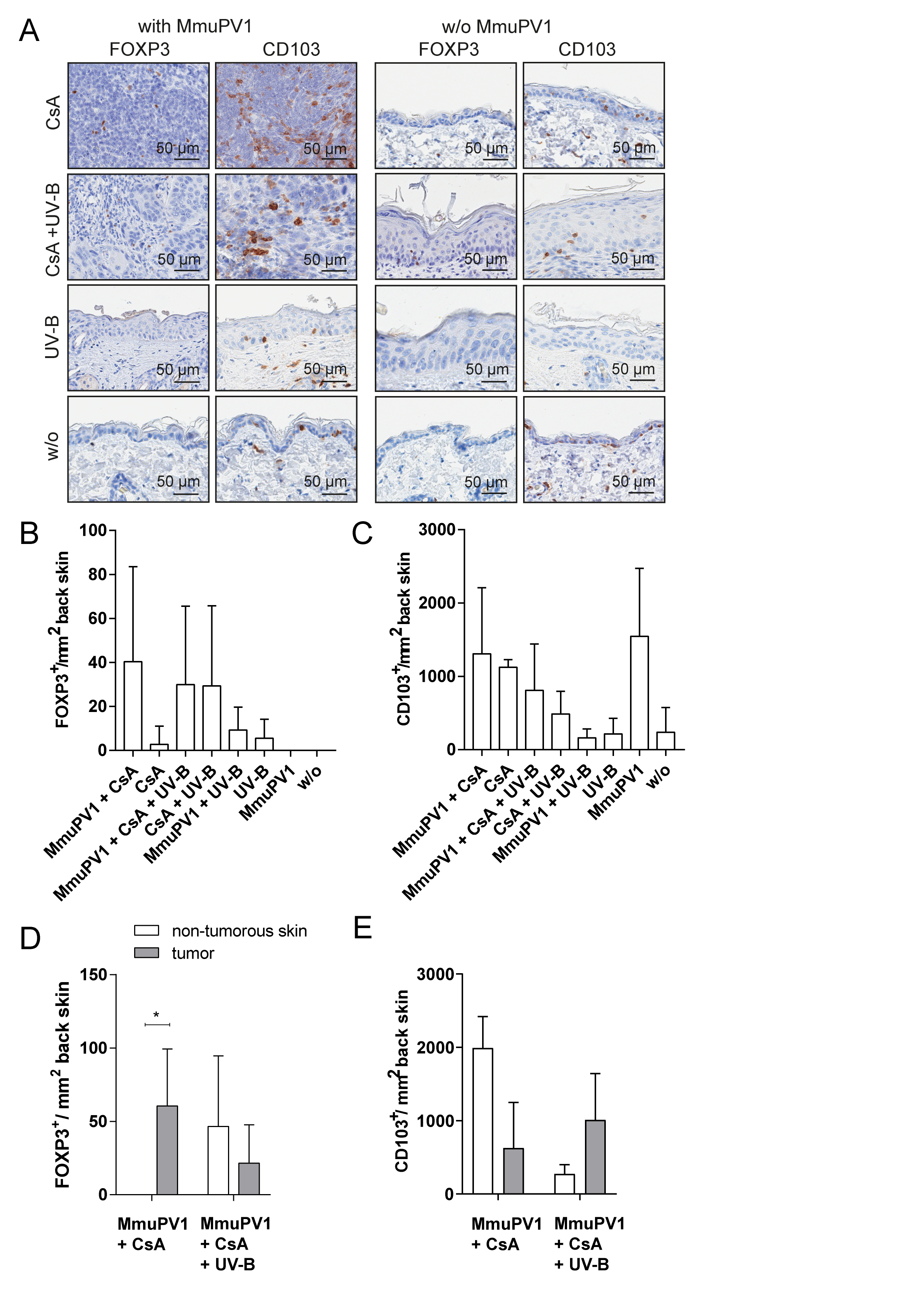

Supplement: Supplementary file 5 — Figure 5: FOXP3+ and CD103+ T‐cells in back skin. A) Representative FOXP3+ (far left panel) and CD103+ (left panel) stainings of MmuPV1‐infected mice. Representative FOXP3+ (right panel) and CD103+ (far right panel) stainings of uninfected control mice. B) Quantification of FOXP3+‐immunopositive T‐cells in back skin; immunopositive T‐cells are given in numbers per mm2 back skin. C) Quantification of CD103+‐immunopositive T‐cells in back skin; immunopositive T‐cells are given in numbers per mm2 back skin. D) Quantification of FOXP3+‐immunopositive T‐cells in tumorous and non‐tumorous skin of MmuPV1‐infected, CsA‐treated and MmuPV1‐infected, CsA‐/UV‐B‐treated mice. E) Quantification of CD103+‐immunopositive T‐cells in tumorous and non‐tumorous skin of MmuPV1‐infected, CsA‐treated and MmuPV1‐infected, CsA‐/UV‐B‐treated mice. [file AJT-21-525-s005.tif]

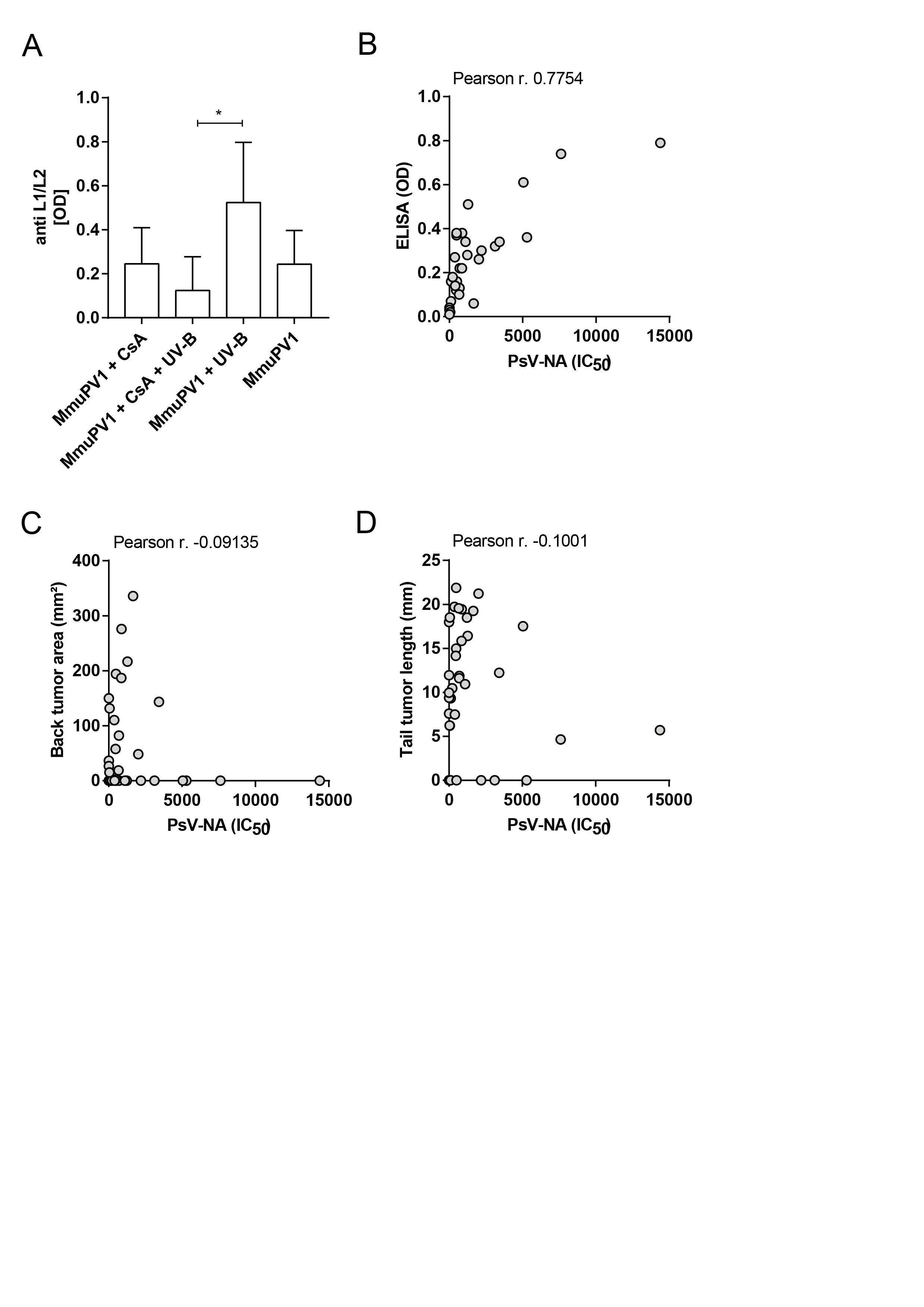

Supplement: Supplementary file 6 — Figure 6: MmuPV1‐specific antibodies in mouse sera. A) MmuPV1‐specific antibodies were determined by particle‐ELISA. B) Correlation of MmuPV1‐specific antibodies with neutralizing antibodies. C) Correlation of MmuPV1‐neutralizing antibodies with back tumor area D) Correlation of MmuPV1‐neutralizing antibodies with tail tumor length. [file AJT-21-525-s006.tif]

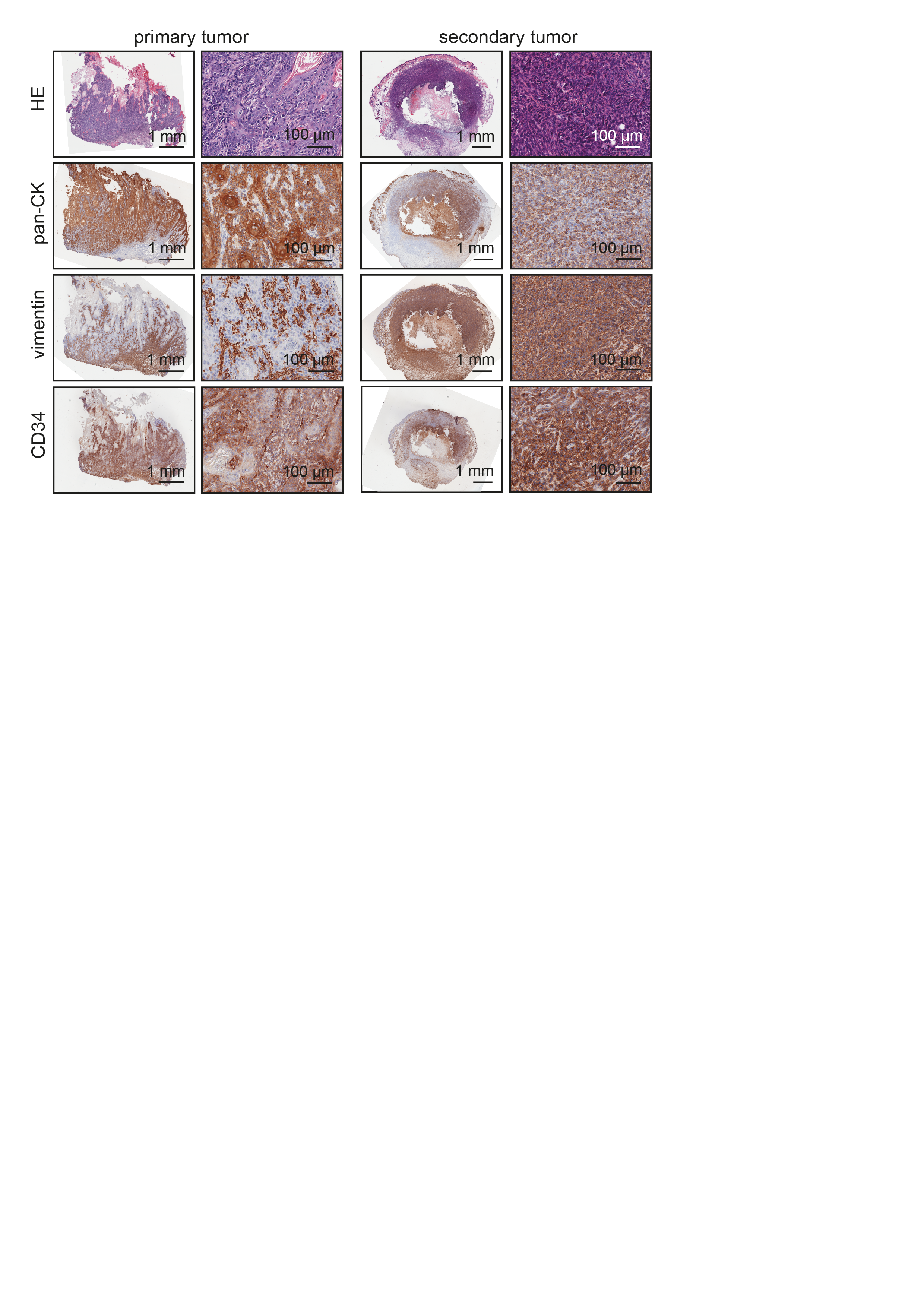

Supplement: Supplementary file 7 — Figure 7: Comparison of pan‐cytokeratin, vimentin and CD34‐staining of primary and secondary cSCCs. Left side: cSCC induced on back skin by MmuPV1 infection in a CsA‐/UV‐B‐treated mouse. Right side: Secondary cSCC which had developed after administration of primary cSCC cells into a NMRIFoxn1nu/nu mouse. The corresponding HE staining of the cSCCs is depicted in the first row. [file AJT-21-525-s007.tif]
