# Supplementary material for: Mus musculus papillomavirus 1 is a key driver of skin cancer development upon immunosuppression
Source: Am J Transplant. 2020 Nov 3;21(2):525–39. doi: 10.1111/ajt.16358 (PMC7894140; doi:10.1111/ajt.16358)
Supplement: Supplementary file 8 [file AJT-21-525-s008.docx]

**Supplementary Table 1: Primary antibodies used for IHC staining.**

| **Antibody** | **Host, isotype** | **Company** | **Clone** | **Dilution** |
| --- | --- | --- | --- | --- |
| **L1/L2** | Rabbit | Eurogentech | polyclonal | 1:4000 |
| **Pan-Cytokeratin** | Rabbit IgG | Abcam | polyclonal | 1:200 |
| **Vimentin** | Rabbit IgG | Abcam | EPR3776 | 1:500 |
| **CD31** | Rabbit IgG | Cell Signaling Technology | D8V9E | 1:100 |
| **CD34** | Rabbit IgG | Abcam | EP373Y | 1:50 |
| **Phospho-Histone H2A.X (Ser 139)** | Rabbit IgG | Cell Signaling Technology | 20E3 | 1:300 |
| **Thymine Dimer**  **(CPD)** | Mouse IgG1 | Kamiya Biomedical Company | KTM53 | 1:500 |
| **BAK** | Rabbit IgG | Cell Signaling Technology | D4E4 | 1:400 |
| **CD4** | Rat IgG1, kappa | Thermo Fisher Scientific | 4SM95 | 1:100 |
| **CD8a** | Rat IgG2a, lambda | Thermo Fisher Scientific | 4SM15 | 1:200 |
| **FOXP3** | Rat IgG2a, kappa | Thermo Fisher Scientific | FJK-16s | 1:100 |
| **CD103** | Rabbit IgG | Abcam | ERP22590-27 | 1:1000 |
| **CD45R/B220** | Rat IgG2a, kappa | BD | RA3-6B2 | 1:500 |
