# Supplementary material for: Mus musculus papillomavirus 1 is a key driver of skin cancer development upon immunosuppression
Source: Am J Transplant. 2020 Nov 3;21(2):525–39. doi: 10.1111/ajt.16358 (PMC7894140; doi:10.1111/ajt.16358)
Supplement: Supplementary file 9 [file AJT-21-525-s009.docx]

**Supplementary Figure 1: Experimental set-up.**

Immunocompetent FVB/NCrl mice were infected with 1x10^10^ MmuPV1 virions per site on the back and tail skin on day 0. CsA treatment and UV-B irradiation were started one week prior to infection. CsA was administered subcutaneously at a dose of 75 mg/kg body weight 5 times per week for the first eleven weeks and subsequently 3 times per week until end of experiment in week 30 post-infection. UV-B irradiation was performed 3 times per week with a starting dose of 120 mJ/cm^2^. The UV-B dose was increased weekly until the final dose of 450 mJ/cm^2^ was reached in week 20 post-infection. Irradiation was continued with the final dose until week 30 post-infection.

**Supplementary Figure 2: Tumor incidence after experimental MmuPV1 skin infection on tail skin.**

**A)** Tumor incidence on tail skin in MmuPV1-infected mice at week 30 post-infection. Uninfected mice did not develop skin tumors.

**B)** Time course of tumor outgrowth on tail skin. Tumor length is given in mm.

**C)** Representative mouse of each experimental group with corresponding HE image. Left panel: MmuPV1-infected, right panel: uninfected mice.

**Supplementary Figure 3: Viral presence in tumors on back skin.**

**A)** Left panel: lower magnification of E6/E7 mRNA present in representative cSCCs of the back. Right panel: corresponding HE stainings.

**B)** Absence of E6/E7 mRNA in infected, adjacent non-tumorous back skin tissues.

**Supplementary Figure 4: Quantification of yH2AX and CPD staining of back skin.**

**A)** Quantification of yH2AX-immunopositivity in tumorous and non-tumorous skin of MmuPV1-infected, CsA-treated and MmuPV1-infected, CsA-/UV-B-treated mice.

**B)** Representative IHC staining for CPD of tumorous and non-tumorous skin.

**C)** Quantification of CPD-immunopositivity in tumorous and non-tumorous skin of MmuPV1-infected, CsA-treated and MmuPV1-infected, CsA-/UV-B-treated mice.

**Supplementary Figure 5: FOXP3^+^ and CD103^+^ T-cells in back skin.**

**A)** Representative FOXP3^+^ (far left panel) and CD103^+^ (left panel) stainings of MmuPV1-infected mice. Representative FOXP3^+^ (right panel) and CD103^+^ (far right panel) stainings of uninfected control mice.

**B)** Quantification of FOXP3^+^-immunopositive T-cells in back skin; immunopositive T-cells are given in numbers per mm^2^ back skin.

**C)** Quantification of CD103^+^-immunopositive T-cells in back skin; immunopositive T-cells are given in numbers per mm^2^ back skin.

**D)** Quantification of FOXP3^+^-immunopositive T-cells in tumorous and non-tumorous skin of MmuPV1-infected, CsA-treated and MmuPV1-infected, CsA-/UV-B-treated mice.

**E)** Quantification of CD103^+^-immunopositive T-cells in tumorous and non-tumorous skin of MmuPV1-infected, CsA-treated and MmuPV1-infected, CsA-/UV-B-treated mice.

**Supplementary Figure 6: MmuPV1-specific antibodies in mouse sera.**

**A)** MmuPV1-specific antibodies were determined by particle-ELISA.

**B)** Correlation of MmuPV1-specific antibodies with neutralizing antibodies.

**C)** Correlation of MmuPV1-neutralizing antibodies with back tumor area

**D)** Correlation of MmuPV1-neutralizing antibodies with tail tumor length.

**Supplementary Figure 7: Comparison of pan-cytokeratin, vimentin and CD34-staining of primary and secondary cSCCs.**

Left side: cSCC induced on back skin by MmuPV1 infection in a CsA-/UV-B-treated mouse. Right side: Secondary cSCC which had developed after administration of primary cSCC cells into a NMRIFoxn1^nu/nu^ mouse. The corresponding HE staining of the cSCCs is depicted in the first row.
